# Deciphering the prognostic features of bladder cancer through gemcitabine resistance and immune-related gene analysis and identifying potential small molecular drug PIK-75

**DOI:** 10.1186/s12935-024-03258-9

**Published:** 2024-04-03

**Authors:** Tingting Cai, Tao Feng, Guangren Li, Jin Wang, Shengming Jin, Dingwei Ye, Yiping Zhu

**Affiliations:** 1 Department of Urology, Fudan University Shanghai Cancer Center, Shanghai, China; 2 Department of Oncology, Shanghai Medical College, Fudan University, Shanghai, China; 3 Department of Urology, The First Affiliated Hospital of Shandong First Medical University, Shandong, China; 4 Shandong Provincial Qianfoshan Hospital, Shandong, China

**Keywords:** Bladder cancer, Prognostic signature, Machine learning, RAC3, PIK-75

## Abstract

**Background:**

Bladder cancer (BCa) stands out as a prevalent and highly lethal malignancy worldwide. Chemoresistance significantly contributes to cancer recurrence and progression. Traditional Tumor Node Metastasis (TNM) stage and molecular subtypes often fail to promptly identify treatment preferences based on sensitivity.

**Methods:**

In this study, we developed a prognostic signature for BCa with uni-Cox + LASSO + multi-Cox survival analysis in multiple independent cohorts. Six machine learning algorithms were adopted to screen out the hub gene, RAC3. IHC staining was used to validate the expression of RAC3 in BCa tumor tissue. RT-qPCR and Western blot were performed to detect and quantify the mRNA and protein levels of RAC3. CCK8, colony formation, wound healing, and flow cytometry analysis of apoptosis were employed to determine cell proliferation, migration, and apoptosis. Molecular docking was used to find small target drugs, PIK-75. 3D cell viability assay was applied to evaluate the ATP viability of bladder cancer organoids before and after PIK-75 treated.

**Results:**

The established clinical prognostic model, GIRS, comprises 13 genes associated with gemcitabine resistance and immunology. This model has demonstrated robust predictive capabilities for survival outcomes across various independent public cohorts. Additionally, the GIRS signature shows significant correlations with responses to both immunotherapy and chemotherapy. Leveraging machine learning algorithms, the hub gene, RAC3, was identified, and potential upstream transcription factors were screened through database analysis. IHC results showed that RAC3 was higher expressed in GEM-resistant BCa patients. Employing molecular docking, the small molecule drug PIK-75, as binding to RAC3, was identified. Experiments on cell lines, organoids and animals validated the biological effects of PIK-75 in bladder cancer.

**Conclusions:**

The GIRS signature offers a valuable complement to the conventional anatomic TNM staging system and molecular subtype stratification in bladder cancer. The hub gene, RAC3, plays a crucial role in BCa and is significantly associated with resistance to gemcitabine. The small molecular drug, PIK-75 having the potential as a therapeutic agent in the context of gemcitabine-resistant and immune-related pathways.

**Supplementary Information:**

The online version contains supplementary material available at 10.1186/s12935-024-03258-9.

## Introduction

Bladder cancer (BCa) stands as the second most frequently diagnosed urinary malignancy, bearing a substantial burden of morbidity and mortality, resulting in an annual toll exceeding 200,000 lives [1]. Currently, treatment regimens and management for BCa heavily rely on histological type, high or low grade, and TNM stage. Nevertheless, the efficacy of these approaches, as determined by the current grading and staging criteria, leaves much to be desired, with long-term survival rates of BCa patients remaining distressingly low [2].

Gemcitabine is applied for both intravesical instillation for non-muscle invasive bladder cancer (NMIBC) and systemic regimens for MIBC owing to its favorable tolerance [3, 4]. Regrettably, inherent or acquired resistance to gemcitabine (GEM) and subsequent tumor recurrence frequently afflict BCa patients, and the underlying mechanisms governing chemoresistance remain elusive. Mounting evidence suggests that chemotherapy exerts diverse effects on the remodeling of the immune tumor microenvironment (TME) [5–7]. Nevertheless, the extent to which GEM resistance may be linked to the restructuring of the immune TME in BCa remains nascent. Consequently, it is of paramount importance to establish an optimal molecular signature characterized by both chemo-responsiveness and immune-related attributes for accurate risk assessment and the selection of personalized treatment strategies, with the potential to ameliorate both prognostic and clinical outcomes for high-risk BCa patients.

In this study, we attempted to predict the prognosis and therapeutic response of BCa patients by characterizing transcriptome features in GEM-resistant BCa cell lines. Using the GSE91061 dataset, perturbed RNA expression associated with GEM resistance was studied, and differentially expressed immune-related genes in GEM-resistant T24 cell lines were incorporated into a new gene set for subsequent model establishment. Consequently, a 13-gene prognostic signature (referred to as GIRS) was constructed by performing Cox proportional hazards regression analysis and LASSO Cox regression analysis utilizing the TCGA-BLCA training cohort, which was significantly associated with the transcriptomic, chemoresistance, and immune infiltration characteristics of BCa. Furthermore, these prognostic values were further confirmed in two independent validation cohorts. Subsequently, we conducted various functional validations based on this model, including therapeutic effect prediction, immune landscape description, GSVA enrichment analysis, correlation analysis, and subtype analysis. Moreover, we identified the hub gene that contributed most significantly to our signature by simultaneously conducting six machine-learning algorithms and further used molecular docking to find potential small molecule drugs targeting this gene.

## Materials and methods

### Data acquisition

All expression profiles and clinical annotations of microarray cohorts were acquired from the GEO database (https://www.ncbi.nlm.nih.gov/geo/). The TCGA-BLCA cohort, along with corresponding clinical characteristics, was retrieved from UCSC Xena (https://tcga.xenahubs.net) or the Supplementary table from Robertson et al. [8]. The IMvigor410 dataset was downloaded using the R package “IMvigor210CoreBiologies” [9]. Somatic mutation and copy number alteration (CNA) information for BCa were obtained from the cBioportal database (http://www.cbioportal.org).

GSE190636 was adopted to obtain the differential expressed genes (DEGs). The TCGA-BCLA, GSE13507, and GSE32894, which contain available survival and clinicopathologic statistics (age, gender, T staging, grade), were applied for the construction and external validation of our novel signature. In addition, two immunotherapy datasets (GSE91061; IMvigor410) and a chemotherapy dataset (GSE52219) were employed to investigate the predictive power of our signature on therapeutic efficiency.

All transcriptome expression profiles were normalized and converted to log2 format. For microarrays, we mapped probe IDs to gene symbols using the corresponding platform comment files. In cases where genes were overlapped by multiple probes, we selected a single probe to represent the gene randomly. For RNA-seq data, the Fragments Per Kilobase of transcript per Million mapped reads (FPKM) values were transformed to Transcripts Per Million (TPM) to ensure a reasonable comparison.

### Identification of gemcitabine-based immune-related genes (GIRGs)

In GSE190636, differentially expressed genes (DEGs) between GEM-resistant T24 cell lines and GEM-sensitive T24 cell lines were identified with a threshold of adj.P.Val < 0.05 and |log2FC|> 1 by using the R package “limma” [10]. The immune-related gene set (IRGs) was predefined and collected from the ImmPort database (https://immport.niaid.nih.gov/). Then, the intersection of DEGs and IRGs was considered as gemcitabine-based immune-related genes (GIRGs) for subsequent analyses. A Venn diagram was adopted to elegantly present the overlapping GIRGs.

### Establishment and validation of gemcitabine-based immune-related risk score (GIRS)

To develop the optimal prognosis signature, we implemented univariate Cox proportional hazards regression analysis (uni-Cox), the least absolute shrinkage and selection operator (LASSO) Cox regression analysis, and multivariate Cox regression analysis (multi-Cox) with “survival” [11] and “glmnet” [12] R package. The regression coefficients obtained from multi-Cox were used to determine the Gemcitabine-based Immune-related Risk Score (GIRS) for each patient via the “survival” R package [11]. Specifically, the GIRS formula was calculated as follows:

$${\mathbf{GIRS}} = \sum {\mathbf{i}}\, {\mathbf{Coefficient}}\left( {\mathbf{i}} \right) \times {\mathbf{Expr}}\left( {\mathbf{i}} \right)$$

where Expr (i) represents the expression level of the gene in patient i, and Coefficient (i) is the multi-Cox coefficient corresponding to gene i. To be more specific, GIRS was calculated as follows: expression of OAS1 * (− 0.116582176) + expression of AHNAK * 0.209856412 + expression of LTBP1 * 0.124776682 + expression of RAC3 * 0.218453203 + expression of GBP2 * − 0.23894607 + expression of SHC3 * (− 0.240912651) + expression of NFATC1 * 0.166421191 + expression of GIPR * (− 0.227052796) + expression of PTK2B * (− 0.23141687) + expression of PAK6 * 0.645350893 + expression of RLN2 * (− 0.219819016) + expression of NAMPT * 0.161238622 + expression of IGF2 * 0.080204401.Patients were categorized into high-risk or low-risk group at the median GIRS for subsequent analyses. The same formula and cut-off value were applied in GSE32894 and GSE13507 for further validation.

To verify the accuracy of the GIRS, the Kaplan–Meier (K–M) survival analysis was conducted using the “survival” [11] and “survminer” [13] R package. The ROC curves (1-,3-,5-year) were generated with the “timeROC” R package [14] and C-index was determined by the R package “survcomp” [15].

### Genomic landscape, immune infiltration, and enrichment analysis between GIRS subgroups

To explore genomic features of GIRGs in BCa, we utilized the “maftools” R package [16] to summarize mutation annotation information The results were visualized into an OncoPrint plot using the “ComplexHeatmap” R package [17].

The Immune cell abundance analysis was conducted by CIBERSORT [18]. Functional enrichment analysis between GIRS subtypes was evaluated using the “GSVA” and “limma” packages based on “hallmark gene sets” and the known oncogenic signaling pathways [19]. 50 hallmark gene sets were extracted from the MsigDB database using the R package “msigdb” [20]. 10 carcinogenic signaling pathways, including HIPPO, NOTCH, PI3K, MYC, RTK-RAS, TGF-beta, NRF2, TP53, Cell cycle, and WNT were collected from previous researches [21] and listed in Additional file 1: Table S3. The oncogenic pathways inhibited or activated in GIRS subgroups separately were determined with t-values > 1 and p < 0.05 as the threshold. The “corrplot” R package [22] was used to explore the association between GIRS and immune cell abundance.

### Cluster analysis, drug sensitivity, CMAP analysis

Using the expression profiles of DEGs, we estimated a clustering analysis in TCGA-BLCA with 3 algorisms (non-negative matrix factorization (NMF), consensus clustering (CC), and similarity network fusion plus consensus clustering (SNFCC+)) by “CancerSubtype” R package [23, 24].

We adopted the R package “pRRophetic” [25] to predict the semi-inhibitory concentration (IC50) of chemotherapeutics for BCa patients to compare their sensitivity within the GIRS subgroup. Potential small molecule drugs were forecasted using the Connectivity Map database (CMap) (https://portals.broadinstitute.org/cmap/).

### Hub gene, transcription factors (TFs) identification

To further pick out the hub gene that contributed most significantly to our model, six machine-learning algorithms (XGboost, Catboost, Random Forest, AdaBoost, LightGBM, GradienBoosting) were employed to analyze the feature importance of the 13 genes in the signature. We utilized SHAP values to interpret the contribution of each gene to the model. SHAP values are an interpretive method used to measure the contribution of each feature to the model output. For each gene, we computed its average SHAP value across all samples to identify genes that significantly contribute to the overall model output.

Otherwise, upstream transcription factors (TFs) that were most likely to regulate the expression of the hub gene were forecasted by Cistrome Data Browser (http://dbtoolkit.cistrome.org/).

### Molecular docking

The molecular docking simulation procedure was employed with the Lamarckian genetic algorithm to explore the correlation between RAC3 and small molecules. The protein crystal structures of RAC3 were gained from the RCSB Protein Data Bank (https:/www.rscb.org/pdb) [26]. The three-dimensional structures of all target compounds were achieved from the PubChem database (https://pubchem.ncbi.nlm.nih.gov/). The small molecule compounds were imported into AutoDock Tools-1.5.6 software, and atomic charges and types were added. All flexible bonds were set to be rotatable, and the structures were saved as pdbqt files. Ten processed compounds were used as small molecule ligands, and two protein targets were used as receptors. The Grid Box’s center position and dimensions were set to 40 × 40 × 40 based on the interaction between small molecules and targets. Molecular docking was performed using AutoDock Vina. The visualization of compound-protein binding interactions was carried out using Pymol 2.1 software. The Lamarckian genetic algorithm was employed for molecular docking calculations, with the following parameters: a population size of 150, a maximum of 25 million energy evaluations, a maximum number of generations set to 2000, a crossover rate of 0.8, a mutation rate of 0.02, 10 independent docking runs, and final docking structures evaluated based on binding free energy. Docking scores were considered in conjunction with interaction patterns to infer the potential activity of screened compounds.

### Tissue digestion and patient derived organoid establishment

Three BCa tissue specimens were collected from patients undergoing transurethral cystectomy (TUR-B) at the Department of Urology, Affiliated Cancer Hospital of Fudan University, to establish Patient-Derived Organoids (PDOs). Tissue specimens were initially placed in a basal medium (Advanced DMEM F12 Serum Free medium, Gibco, 12634010). Following mechanical disruption, the tumor tissues were minced into a paste, washed in Basal medium (800×*g* for 5 min), and then digested at 37 °C in an enzyme mixture containing 2.5 mg/ml Collagenase II (ThermoFisher 17101015) and 10 μM Y-27632-HCl Rock Inhibitor (MCE, 146986–50-7) in Basal medium for 10–15 min, with stirring every 5 min. The digested tissue was further washed with Basal medium (1000 rpm, 5 min). The resulting precipitate was resuspended in 1–2 ml TrypLE Express (ThermoFisher, 12605028) and digested at 37 °C for 5 min. The digestion process was halted with Basal medium through a 70 μm cell strainer (Corning, 352350). The cell filtrate was centrifuged again (1000 rpm, 5 min). Cell clusters were then resuspended in organoid matrix gel (Coring, 356231) and seeded into Ultra Low Adhesion (ULA) 24-well plates (Coring, 3743). After a 30 min incubation at 37 °C under 5% CO2 conditions, Bladder Cancer Organoid Specific Medium (500 μl/well, Bladder Organoid Kit, K2126-CB) was added. CellTiter-Glo 3D (Promega, G9682) was employed to measure relative cell activity following the manufacturer’s instructions.

### Immunohistochemistry (IHC) staining of RAC3

IHC was performed on FFPE sections. The antibody used was RAC3 (1:200, ab124943, Abcam). The paraffin specimens were obtained from 10 patients diagnosed with NMIBC at our center in the year 2023. Each patient underwent transurethral resection of bladder tumor (TURBT) followed by intravesical gemcitabine instillation.

### Cell culture and transfection

Bladder cancer cell lines T24 and 5637 were obtained from the American Type Culture Collection (ATCC) and cultured in DMEM supplemented with 10% FBS and 100 U/ml penicillin–streptomycin at 37 °C with 5% CO2. RAC3 knockdown plasmids, shRAC3, and control empty vector shNC, were transfected into the bladder cancer cells using Lipofectamine 3000. The shRNA sequences were provided in Additional file 1: Table S5.

### RNA extraction and reverse transcriptase quantitative-polymerase chain reaction(RT-qPCR) and western blot

The primers of RAC3 (F: TCCCCACCGTTTTTGACAACT; R: GCACGAACATTCTCGAAGGAG), GAPDH (F: CTGGGCTACACTGAGCACC; R: AAGTGGTCGTTGAGGGCAATG) were designed using the primer 5.0. Total RNAs were extracted using an RNA Extraction Kit (Nuoweizan Biotechnology, Nanjing, China), and were reversely transcribed into cDNA using PrimeScript RT Master Mix (Takara, Shiga, Japan). The RNA expression levels of RAC3 were quantified using the 2^–ΔΔCT^ (Livak) method.

Western blotting was performed as previously described. Proteins were extracted from harvested BCa cells separately and quantified by BCA assay. The primary antibodies used in this study were recombinant anti-RAC3 antibody (1:1000, ab124943, Abcam) and anti-GAPDH antibody (1:5000, ab181602, Abcam) in our study.

### Cell proliferation and migration assay

CCK-8 and colony formation assay were used for the proliferation test. Wound-healing assay were used for the migration test. Covered surface areas were measured using Image-J software.

### Cell apoptosis analysis

Apoptosis was examined by flow cytometry using Annexin V-PE/7-AAD Apoptosis Kit (Liankebio, Hangzhou, China) following the protocol of the instructions. We employed EDTA-free pancreatic enzyme digestion during the experiment to minimize cell damage from digestion and pipetting, thus reducing potential experimental biases. After 24 h treatment with PIK-75 or DMSO, cells were harvested and incubated with Annexin V-PE and 7-AAD for 5 min under light-protected, room temperature conditions, followed by immediate analysis by flow cytometry.

### Subcutaneous tumor model

Following the injection of 5 × 10^6^ MB49 cells (100 μl) into six-week-old C57BL/6 mice, tumor volumes were assessed weekly using the formula: volume(mm^3^) = 0.52 × (length × width^2^). After 35 days, all mice were sacrificed. Additionally, the mice were subjected to intraperitoneal administration(i.p.) of PIK-75 or DMSO (as a control) following tumor establishment, to evaluate the therapeutic efficacy of PIK-75 against BCa.

### Statistical analysis

The majority of the statistical analyses were conducted using R software 4.2.1 or GraphPad Prism 6. Otherwise, six machine learning algorithms, XGboost, Catboost, Random Forest, AdaBoost, LightGBM, and GradienBoosting were implemented with Python 3.8.5 version. Differences between variables were ascertained by t-test or one-way ANOVA, respectively. Non-parametric tests (Wilcoxon test and Kruskal–Wallis tests) were also applied when the homogeneity of variance was not satisfied. Correlation analysis among variables was conducted by Pearson or Spearman analysis. All P values were bilateral, and P < 0.05 was considered a statistically significant difference.

## Results

### Identification of gemcitabine-based immune-related genes (GIRGs) in BCa

**Fig. 1:**
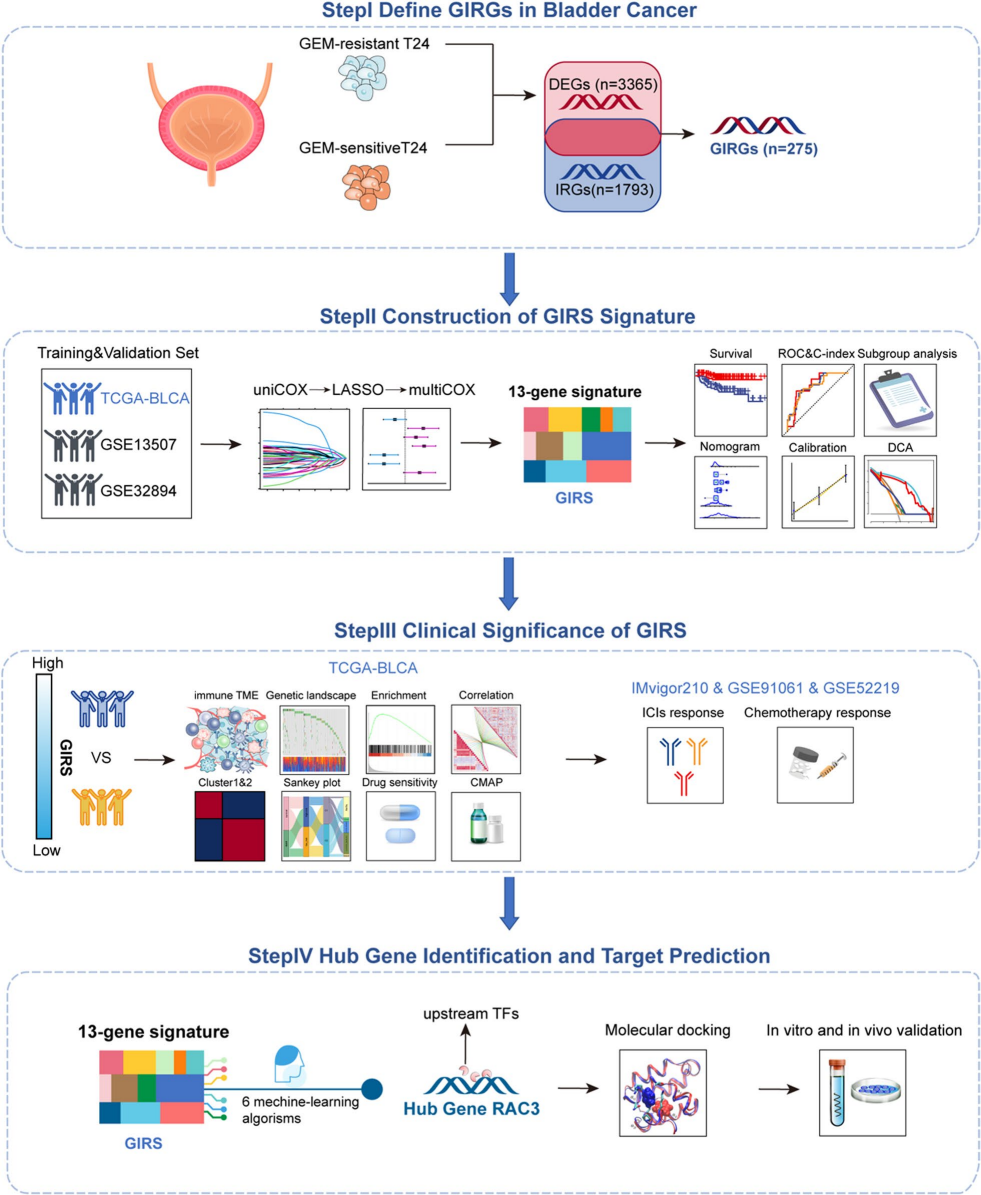
The workflow of our study

**Fig. 2:**
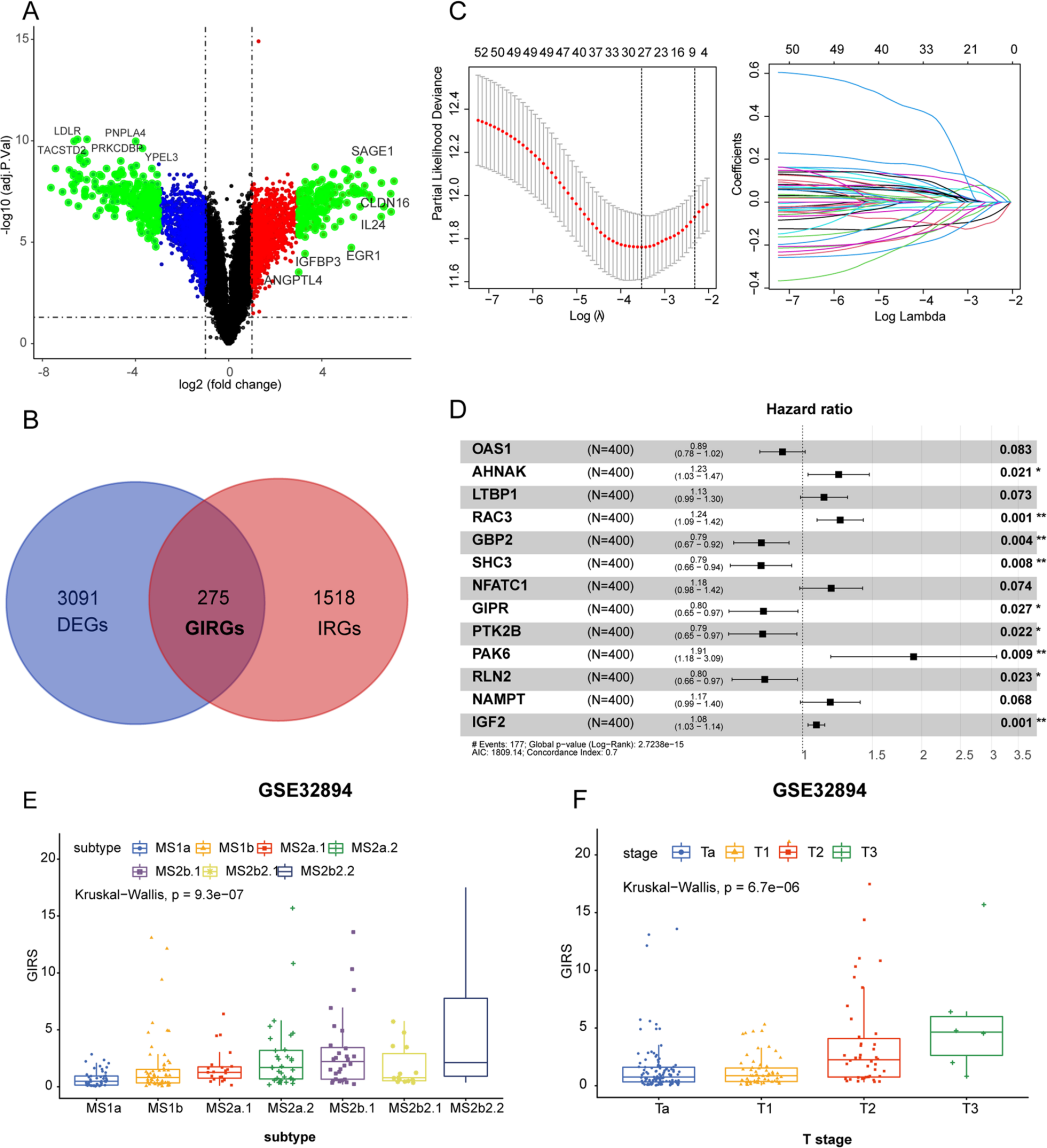
Identification of GIRGs and construction of GIRS signature. **A** A volcano plot was used to visualize the DEGs between GEM-sensitive and GEM-resistant T24 cell lines. **B** A Venn diagram was adopted to visualize the GIRGs. **C** The LASSO regression lambda filter. The optimal λ was generated when the partial likelihood of deviance reached the minimum value and the corresponding LASSO coefficients of each GIRG were also obtained. **D** The forest plot illustrated the Multivariate Cox regression analysis of OS in the TCGA training cohort. **E, F** GIRS scores among distinct molecular subtypes [Lund1 staging system (**E**); AJCC TNM staging system (**F**)] in GSE32894. Statistical comparisons were conducted using the Kruskal–Wallis test

The workflow of our study was shown in Fig. 1. The Gemcitabine-based Immune-Related Genes (GIRGs) gene set was delineated by analyzing differentially expressed genes (DEGs) in gemcitabine-resistant and sensitive cell lines, followed by the intersection with immune-related genes (IRGs). Specifically, a total of 3365 DEGs, comprising 1691 upregulated and 1674 downregulated genes, were identified (Fig. 2A). Subsequent intersection with IRGs resulted in the identification of 275 GIRGs (Fig. 2B and Additional file 1: Table S1). So, we posited that GIRGs were intricately associated with the immune mechanisms underlying gemcitabine resistance in BCa. This gene set served as the foundation for the subsequent development of clinical prognostic signature.

### Genetic landscape of GIRGs in high and low-risk subgroups

Somatic mutations and copy number variants are believed to be involved in cancer ontogeny and may be associated with prognosis, drug sensitivity, and immune phenotype [2], and BCa possesses one of the highest somatic alterations of all cancers. To explore the prospective connection between somatic alterations and GIRS, we analyzed the patterns of gene mutations and copy number variations (CNVs) between the high GIRS and low GIRS subgroups in the TCGA-BLCA cohort. The overall landscapes of somatic mutations per case in the two groups showed that the tumor mutation burden of the high GIRS subgroup was significantly higher than that of the low GIRS subgroup. The range of mutations per case in the high-risk group varied from 0 to 37, while in the low-risk group, it ranged from 0 to 19 (Additional file 1: Fig. S2A and C). In terms of specific genes, LRP1 had a relatively high mutation frequency of 10% in the high-risk group, whereas AHNKK (16%) and FGFR3 (14%) had relatively high mutation frequencies in the low-risk group (Additional file 1: Fig. S2A and C). Moreover, the main type of single nucleotide variant (SNV) was Missense_Mutation, and C → T was the primary style of base conversion in both two groups. Thirty genes that possessed a higher alterations frequency were listed in Additional file 1: Fig. S2B and D.

### Establishment and validation of the GIRS signature for the prognostic prediction of bladder cancer

**Fig. 3:**
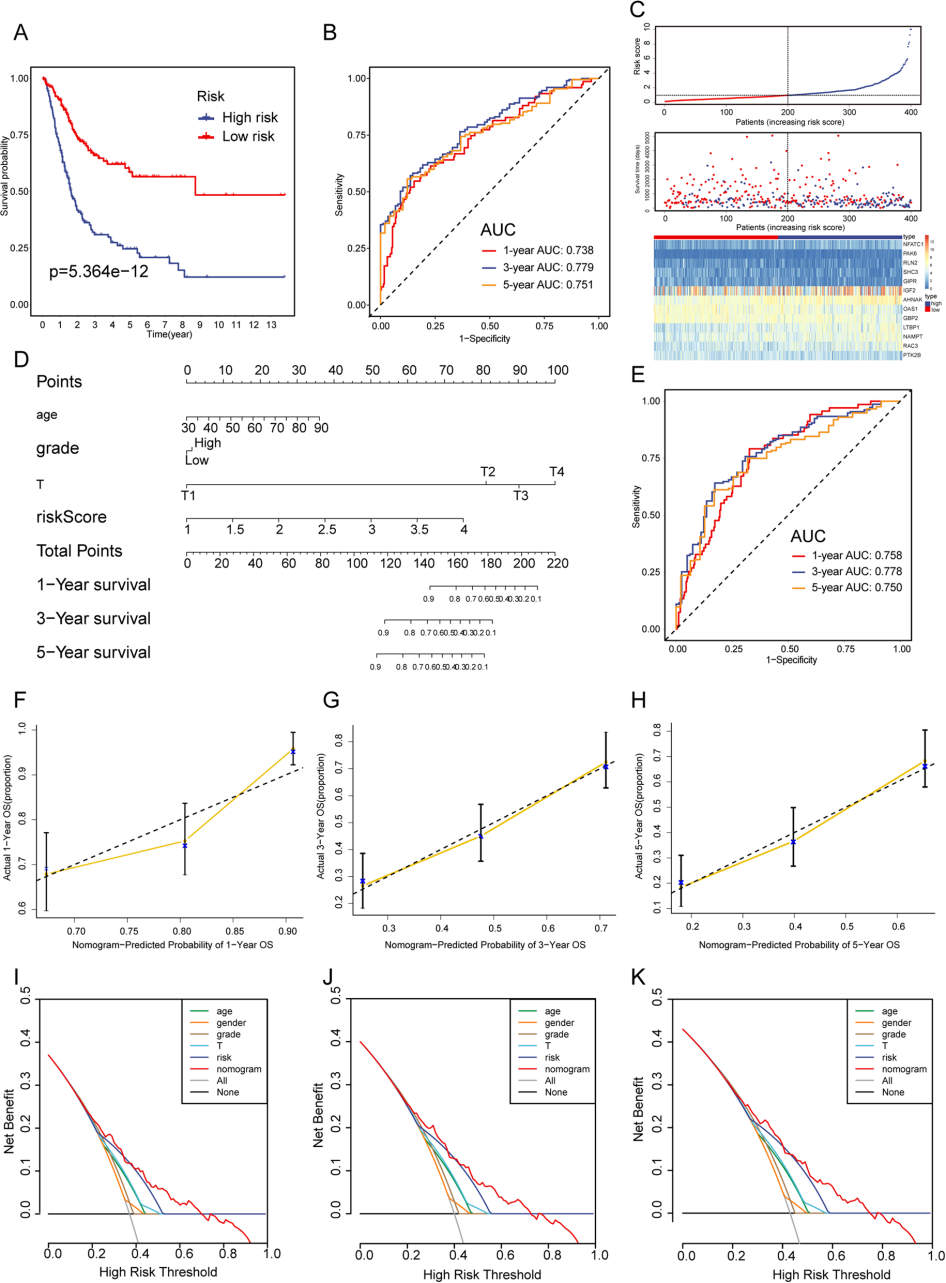
Survival analysis between GIRS subgroups and nomogram development in the TCGA-BLCA training cohort. **A** Kaplan–Meier curve analysis of OS between high and low GIRS subgroups. **B** Time-dependent ROC analysis of GIRS for predicting OS at 1, 3, and 5 years. **C** The distribution of GIRS signature, the vital status of patients, and the expression of GIRGs. **D** Nomogram development. **E** Time-dependent ROC curves at 1, 3, and 5 years of the nomogram. **F**–**H** The calibration plots of nomogram for predicting BCa patients with 1, 3, and 5-year OS. The nomogram’s ideal performance is shown by the dashed lines. **I**–**K** The decision curve analysis of nomogram and other clinical factors for 1, 3, and 5-year risk. The black line represents the hypothesis that no patient died after 1, 3, and 5 years

In the TCGA-BLCA training cohort, the modeling approach employed is a stepwise survival analysis framework, incorporating the univariate Cox analysis (uni-Cox), LASSO-Cox, and multivariate Cox analysis (multi-Cox) models. Firstly, the uni-Cox model provided a preliminary understanding of each gene, assisting in the identification of factors potentially associated with survival time. 57 survival-related GIRGs were screened out (Additional file 1: Table S1). Subsequently, LASSO was utilized for feature selection and regularization, helping to reduce the model's complexity by pinpointing features with significant impacts on survival time. As shown in the results of Fig. 2C, when the partial likelihood deviance reaches its minimum, the optimal λ is determined to be 27 (Fig. 2C; Additional file 1: Table S2). Then, the multi-Cox model considered multiple variables, simultaneously adjusting for their effects on survival time to achieve a more comprehensive understanding of the relative contributions of each factor. Eventually, 13 GIRGs were selected to establish the gemcitabine-based immune-related gene signature (GIRS) and the formula for calculating the risk score is derived from the coefficients obtained in the final step of multi-Cox by the corresponding gene expression level, referred to as GIRS (Fig. 2D), GIRS = expression of OAS1 * (− 0.116582176) + expression of AHNAK * 0.209856412 + expression of LTBP1 * 0.124776682 + expression of RAC3 * 0.218453203 + expression of GBP2 * − 0.23894607 + expression of SHC3 * (− 0.240912651) + expression of NFATC1 * 0.166421191 + expression of GIPR * (− 0.227052796) + expression of PTK2B * (− 0.23141687) + expression of PAK6 * 0.645350893 + expression of RLN2 * (− 0.219819016) + expression of NAMPT * 0.161238622 + expression of IGF2 * 0.080204401. Based on the median value of GIRS, patients can be categorized into high-risk and low-risk groups. The K-M survival analysis showed poorer overall survival in the high GIRS group than in the low GIRS group (Log-rank test, p = 5.364e−12, Fig. 3A). As to the model evaluation, the AUC values for the GIRS model of 1, 3and 5 year were 0.738, 0.779, and 0.751 (Fig. 3B). The C-index of GIRS was 0.7021958 (95% confidence interval (CI) 0.6606−0.7438, p = 1.643737e−21).

**Fig. 4:**
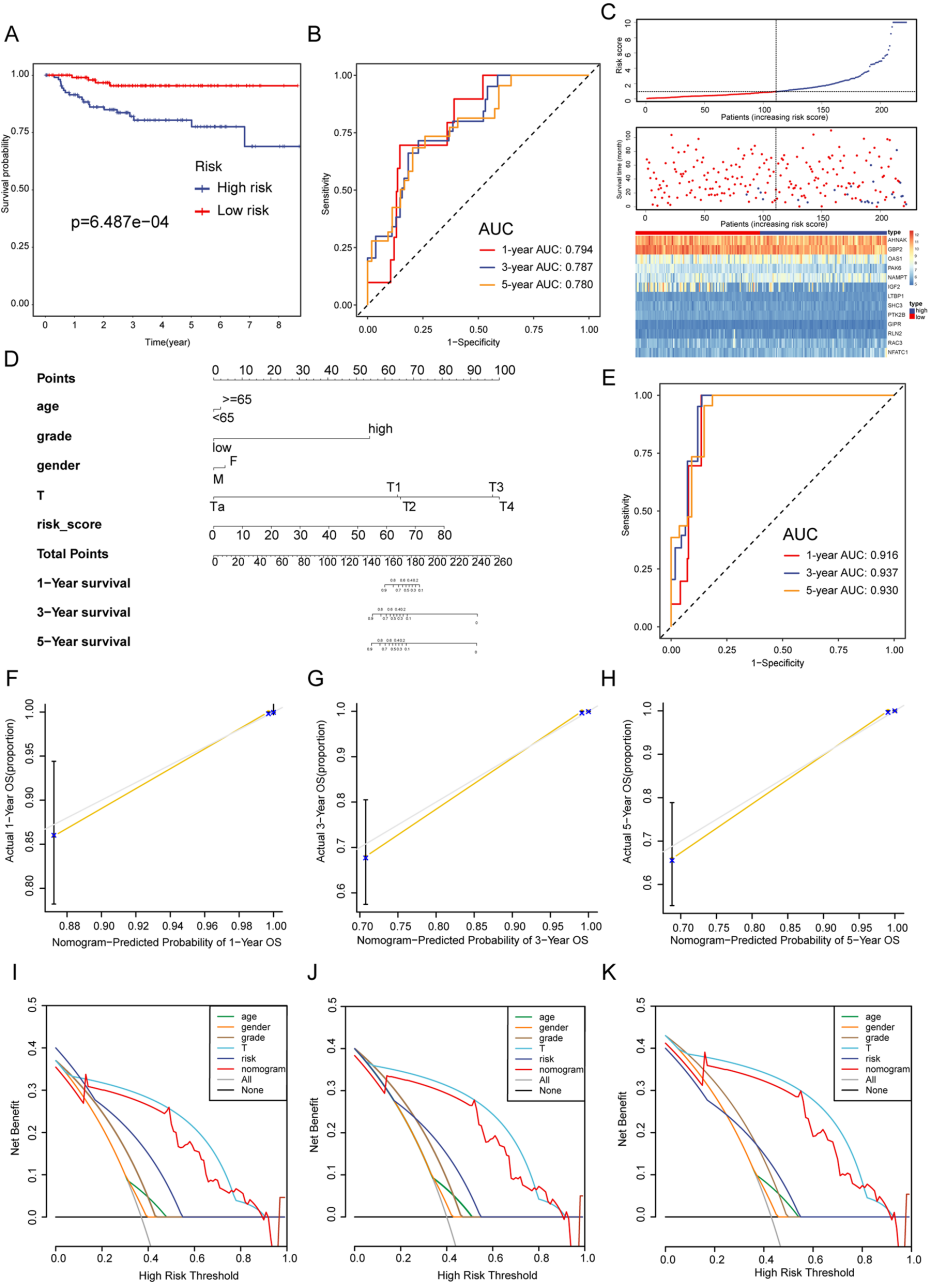
Survival analysis between GIRS subgroups and nomogram validation in the validation cohort GSE32894. **A** Kaplan–Meier curve analysis of OS between high and low GIRS subgroups. **B** Time-dependent ROC analysis of GIRS for predicting OS at 1, 3, and 5 years. **C** The distribution of GIRS signature, the vital status of patients, and the expression of GIRGs. **D** Nomogram development. **E** Time-dependent ROC curves at 1, 3, and 5 years of the nomogram. **F**–**H** The calibration plots of nomogram for predicting BCa patients with 1, 3, and 5-year OS. The nomogram’s ideal performance is shown by the dashed lines. **I**–**K** The decision curve analysis of nomogram and other clinical factors for 1, 3, and 5-year risk. The black line represents the hypothesis that no patient died after 1, 3, and 5 years

To further assess the robustness of the GIRS model, its performance was validated in two independent cohorts (GSE13507, GSE32894). We found that the GIRS worked well in all validation cohorts and that the high GIRS group had poorer OS than the low group (GSE32894, Logrank test, p = 6.487e−04, Fig. 4A; GSE13507, Log-rank test, p = 3.96e−02, Additional file 1: Fig. S1A). Moreover, ROC curves and the C-index showed moderate to high sensitivity and specificity in all validation sets as well. In GSE32894, the AUC values of 1, 3 and 5 year were 0.794, 0.787, 0.780 (Fig. 4B), and the C-index was 0.7968 (95% confidence interval (CI) 0.7201−0.8736, p = 3.391163e−14). In GSE13507, the AUC values of 1, 3 and 5 year were 0.646, 0.562, 0.588 (Additional file 1: Fig. S1B), and the C-index was 0.5953 (95% confidence interval (CI) 0.5216–0.6690, p = 0.0112). Additionally, the distribution of the GIRS, survival time, survival status and the expression of 13 GIRGs in three datasets were visualized in Figs. 3C, 4C, and Additional file 1: Fig. S1C.

Additionally, we conducted a comprehensive comparison between GIRS and established molecular subtypes of BCa. We observed that lower GIRS corresponded to better prognostic subgroups, such as MS1a and MS1b (Kruskal−Wallis, p = 9.3e−07), as well as lower T stage (Kruskal−Wallis, p = 6.7e−06), reflecting the adequate reliability and extrapolation nature of our signature (Fig. 2E and F). These findings underscore the robust reliability and generalizability of our GIRS signature (Fig. 2E and F). The ability of GIRS to align with distinct molecular subtypes of BCa and their corresponding clinical outcomes highlights its potential as a reliable prognostic tool, demonstrating its superiority in characterizing the heterogeneity within BCa.

In summary, our GIRS signature has demonstrated consistent and robust accuracy in prognostic prediction, as evidenced by the results of KM curves, ROC curves, and the C-index in both the training and validation cohorts. In practical clinical scenarios, such as the management of NMIBC patients facing multiple options for intravesical instillation, the treatment decision becomes pivotal. Specifically, for a patient identified with a high GIRS, choosing BCG infusion over gemcitabine or epirubicin may offer more significant clinical benefits.

### Nomogram construction and validation

To further optimize the GIRS and provide a clinically relevant quantitative method for clinicians to predict the prognosis of patients, a nomogram that integrated the GIRS and other clinical parameters was formulated to visualize the risk prediction of survival in 1, 3, or 5 years (Figs. 3D, 4D, and Additional file 1: Fig. S1D). Patients with accessible clinical statistics in the TCGA-BLCA training cohort (N = 365), GSE13507 (N = 164), and GSE32894 (N = 222) validation set were put into the construction of nomograms. Following independent parameters, age, gender, T stage, grade, and GIRS were assigned scores via the “rms” R package [3]. Higher total points on the nomogram corresponded to worse clinical outcomes for patients. Remarkably, the incorporation of the above clinical variables into the nomogram resulted in significantly increased AUC and C-index compared to the GIRS alone. Particularly in GSE32894, the AUC values of 1, 3and 5 year were 0.916, 0.937, 0.930 (Fig. 4E), and the C-index was 0.9262 (95% confidence interval (CI) 0.8994 – 0. 0.9530, p = 2.455099e-213). Similarly, in the TCGA-BLCA cohort, the AUC values of 1, 3a nd 5 year were 0.738, 0.779, 0.751 (Fig. 3E), and the C-index was 0.7195 (95% confidence interval (CI) 0.6805–0.7585, p = 2.497877e−28). In GSE13507, the AUC values of 1, 3and 5 year were 0,683, 0.879, 0.904 (Additional file 1: Fig. S1E), and the C-index was 0.7687 (95% confidence interval (CI) 0.7009–0.8366, p = 8.238777e-15).

Moreover, calibration curves and decision curve analysis (DCA) were employed to visually appraise the consistency of the nomogram and the net clinical benefit with the “rmda” R package [4, 5]. The calibration curves demonstrated a strong alignment between the predicted results and actual survival outcomes, as illustrated in Figs. 3F–H, 4F–H, and Additional file 1: Fig. S1F–H. The nomogram’s ideal performance is shown by the dashed lines. The DCA curves indicated that the nomogram has the highest predictive efficacy compared to other existing criteria, such as T staging, suggesting its substantial potential for clinical application (Figs. 3I–K, 4I–K, and Additional file 1: Fig. S1I–K). Overall, the proposed nomogram demonstrated excellent performance in the TCGA-BLCA training cohort and the validation cohorts, underscoring its potential as a reliable prognostic tool.

### Implications of GIRS for immunotherapy, chemotherapy, and biological mechanisms

**Fig. 5:**
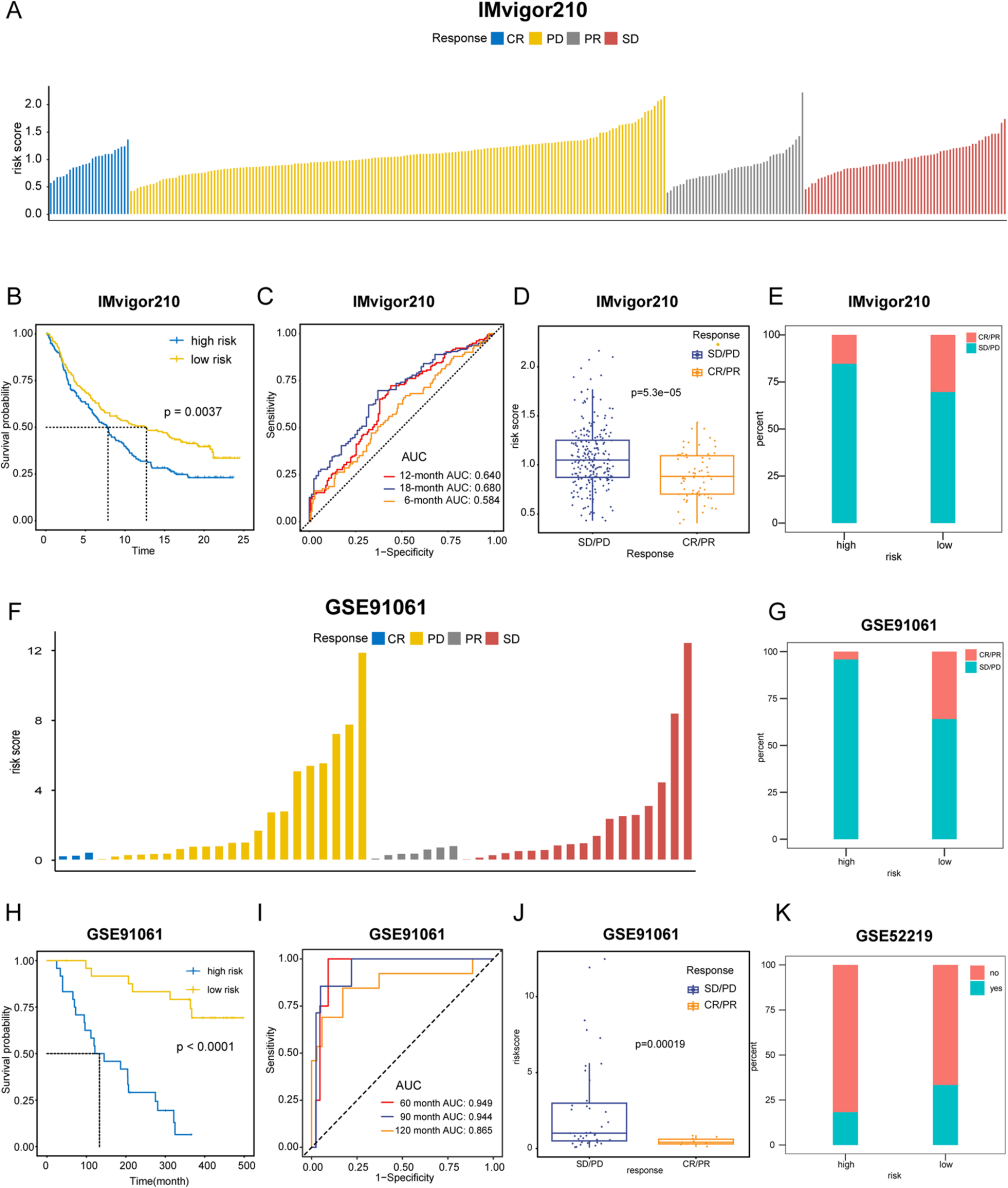
Implications of GIRS for immunotherapy and chemotherapy response prediction in three therapeutic cohorts. **A** Distribution of GIRS for patients exhibiting different immunotherapeutic responses in the IMvigor210. **B** Kaplan–Meier curve analysis of OS between high and low GIRS subgroups in the IMvigor210. **C** Time-dependent ROC analysis for predicting OS in the IMvigor210. **D** GIRS score in the CR/PR group and SD/PD group in IMvigor210. Statistical comparisons were conducted using the t-test (p = 5.3e−05). **E** Boxplot displayed the GIRS signature in patients with different immunotherapy responses in the IMvigor210. **F** Distribution of GIRS for patients exhibiting different immunotherapeutic responses in the GSE91061. **G** Boxplot displayed the GIRS signature in patients with different immunotherapy responses in the GSE91061. **H** Kaplan–Meier curve analysis of OS between high and low GIRS subgroups in the GSE91061. **I** Time-dependent ROC analysis for predicting OS in the GSE91061. **J** GIRS score in the CR/PR group and SD/PD group. Statistical comparisons were conducted using the t-test (p = 0.00019). **K** Boxplot displayed the GIRS signature in patients with different immunotherapy responses in the GSE52219

Due to the high tumor burden and immunogenicity of BCa, immune checkpoint inhibitors (ICIs) are considered promising treatments for BCa. However, due to tumor heterogeneity, only a minority of patients exhibit a positive response. The identification of predictors has been critical to the development of immunotherapy strategies. Hence, our primary objective was to investigate whether the GIRS could serve as a predictor for immunotherapy efficacy. We utilized two immunotherapy-related datasets: the uroepithelial carcinoma dataset (IMvigor210), comprising samples treated with anti-PD-1, and the malignant melanoma dataset (GSE91016), involving samples treated with both anti-PD-1 and anti-CTLA-4. Patients were classified into four categories according to the difference in treatment efficacy: progressive disease (PD), stable disease (SD), partial remission (PR), and complete remission (CR). GIRS was calculated from the expression profile data of the mentioned cohorts, and patients were divided into high and low GIRS subtypes accordingly. The distributions of GIRS for patients exhibiting different immunotherapeutic responses was shown in Fig. 5A and F. The K–M survival analysis showed a higher OS for the low GIRS subgroup in the above two datasets (IMvigor210, Log-rank test, p = 0.0037, Fig. 5B, and GSE91016, Logrank test, p < 0.0001, Fig. 5H), in agreement with the previous training and validation set results, further demonstrating the robustness and extractability of the model in predicting prognosis. As regards the prediction accuracy, the average AUC values of 6-, 12and 18-month prognosis predictions reached 0.640, 0.680, and 0.584 on the IMvigor210 cohort (Fig. 5C). Similarly, in GSE91016, the AUC values of 60-, 120and 180-month were 0.949, 0.944, and 0.865 (Fig. 5I). Then it was observed that a higher percentage of patients responded to ICIs in the low GIRS group, (Fig. 5E and G), and the overall GIRS score in the CR/PR group was lower than that in the SD/PD group (IMvigor210, t-test, p = 5.3e−05, Fig. 5D, and GSE91016, t-test, p = 0.00019, Fig. 5J). The above results implied that patients receiving ICIs had better disease regression in the low-GIRS group than in the high-GIRS group. This may be part of the explanation for why the low-GIRS group exhibited better overall survival than the high-GIRS group. Similarly, in cohort GSE52219, a greater proportion of those classified as low-GIRS responded to MVAC neoadjuvant chemotherapy regimens (Methotrexate; Vinblastine Sulfate; Adriamycin; Cisplatin) (Fig. 5K). In summary, GIRS could serve as a valid biomarker for predicting therapeutic efficacy in ICIs immunotherapy and chemotherapy in BCa patients.

### Enrichment analysis, cluster analysis and correlation analysis between GIRS subtypes

**Fig. 6:**
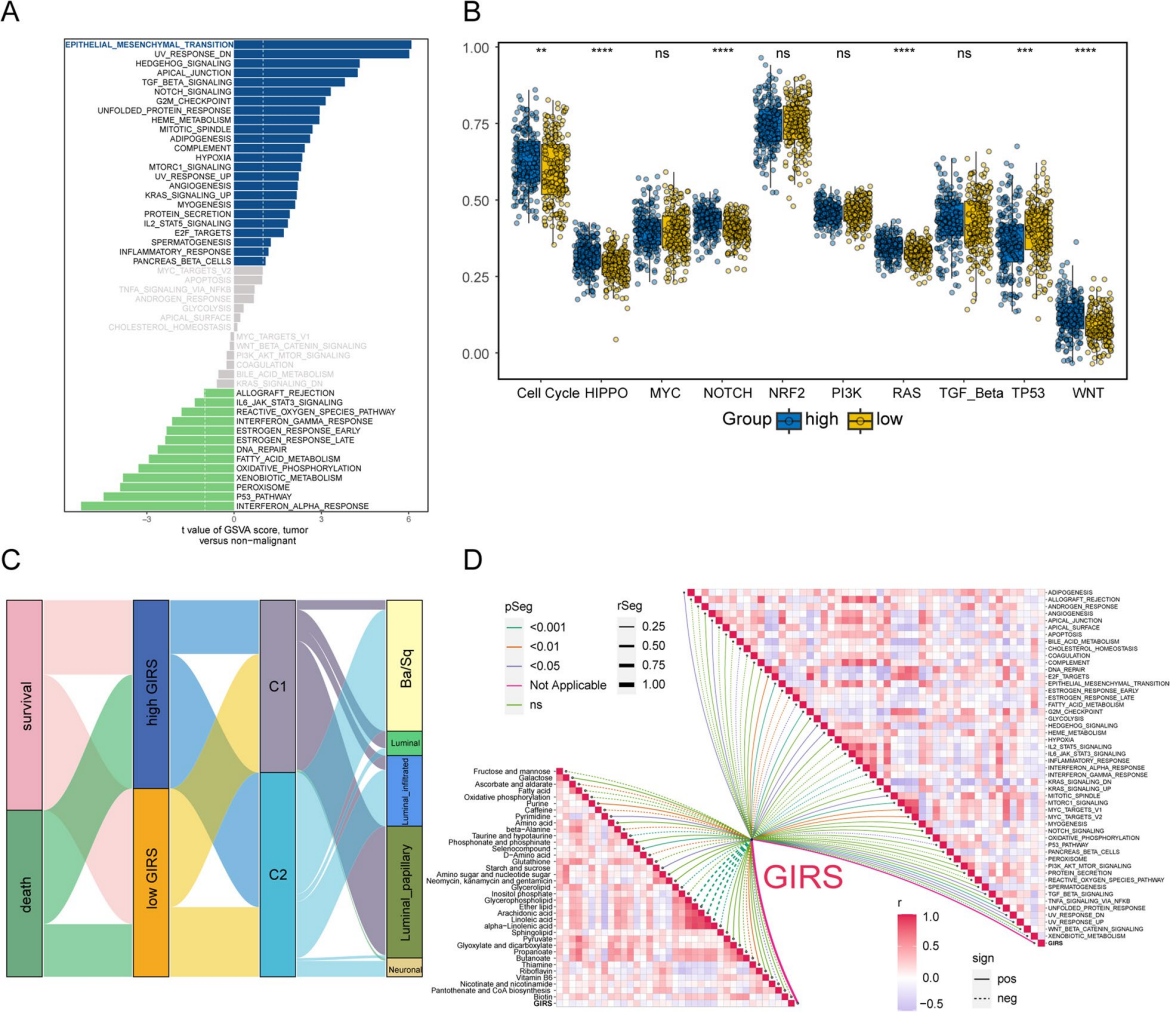
Enrichment analysis and correlation analysis between GIRS subtypes based on the TCGA cohort. **A** Difference in pathway activities scored per patient by GSVA between the high and low GIRS subgroups. Shown were t values from a linear model. **B** Violin plot illustrated the difference in oncopathways between the high and low GIRS subgroups. **C** Sankey plot visualized the relationships among the C1/C2 clusters, Lund1 subtypes, GIRS, and survival status. **D** Butterfly plot illustrated the correlation between the GIRS and metabolic pathways, the enrichment pathways based on GSVA of GO and KEGG terms

The difference in pathway activities scored per patient by GSVA between the high and low GIRS subgroups was shown in Fig. 5A. In high GIRS group, genes related to epithelial to mesenchymal transition (EMT) are significantly enriched (Fig. 6A). Several oncopathways, such as cell cycle-related pathway, HIPPO pathway, NOTCH pathway, RAS pathway, and WNT pathway, were activated in the high GIRS group (Fig. 6B; all p-value < 0.001).

Furthermore, we operated a clustering analysis in the TCGA-BLCA cohort. The results demonstrate that NMF manifests comprehensive advantages where subgroup 1 consists of 183 samples, and subgroup 2 has 217 samples. All three clustering algorithms, including NMF, SNFCC+, and CC, successfully differentiated the patient groups based on clinical survival information (NMF: p = 0.000498; SNFCC+: p = 8.34e−05; CC: p = 3.38e−05) (Additional file 1: Fig. S5C). NMF outperforms the other methods in terms of the average silhouette width (ASW) method (NMF: ASW = 1; SNFCC+: ASW = 0.99; CC: ASW = 0.86) (Additional file 1: Fig. S5B). Additionally, NMF exhibited distinct boundaries between color clusters, representing individual patient communities, indicating accurate and effective isolation of the subgroups. In summary, of these three evaluation methods, NMF more accurately characterized the inter-sample correlations and felicitously isolated the subpopulations. The results of the Sankey diagram in Fig. 6C indicated that subgroup 2 tended to correspond to MIBC subtypes with worse prognosis, such as Ba/Sq subtype, luminal-infiltrated subtype, and neuroendocrine type.

Additionally, as depicted in Fig. 6D, we also carried out a correlation analysis of metabolism-related pathways and GIRS. We could find that GIRS had a negative correlation with lipid metabolism-related pathways.

We then examined the discrepancies in the immune landscape between GIRS subtypes in an attempt to explore possible underlying mechanisms for the differences in treatment efficacy. Since our prognostic gene signature was based on the differential expression of immune-related genes, it was reasonable to assume that GIRS could be closely related to immune infiltration, thereby causing differences in immunotherapeutic efficacy. To confirm this speculation, we performed immune cell infiltration analyses via the CIBERSORT algorithm. The abundance of 24 immune cell types was depicted in Additional file 1: Fig. S3A and C. Moreover, we investigated the diversity in tumor-infiltrating immune cells in the TME. We found that the low-GIRS group had higher CD8 T cell infiltration (Wilcoxon test; p < 0.0001; Additional file 1: Fig. S3B), and GIRS was significantly positively correlated with CD4 memory cells and M1 macrophages (r > 0.4; Additional file 1: Fig. S3D). Hence, we further described 30 types of T cells and macrophages in the high or low GIRS subgroup. Overall, the low GIRS group had a relatively higher infiltration of T cells and macrophages, with significant differences in all types of CD8 T cells in particular (Additional file 1: Fig. S4).

### Drug sensitivity analysis and small-molecule targeted drugs prediction

**Fig. 7:**
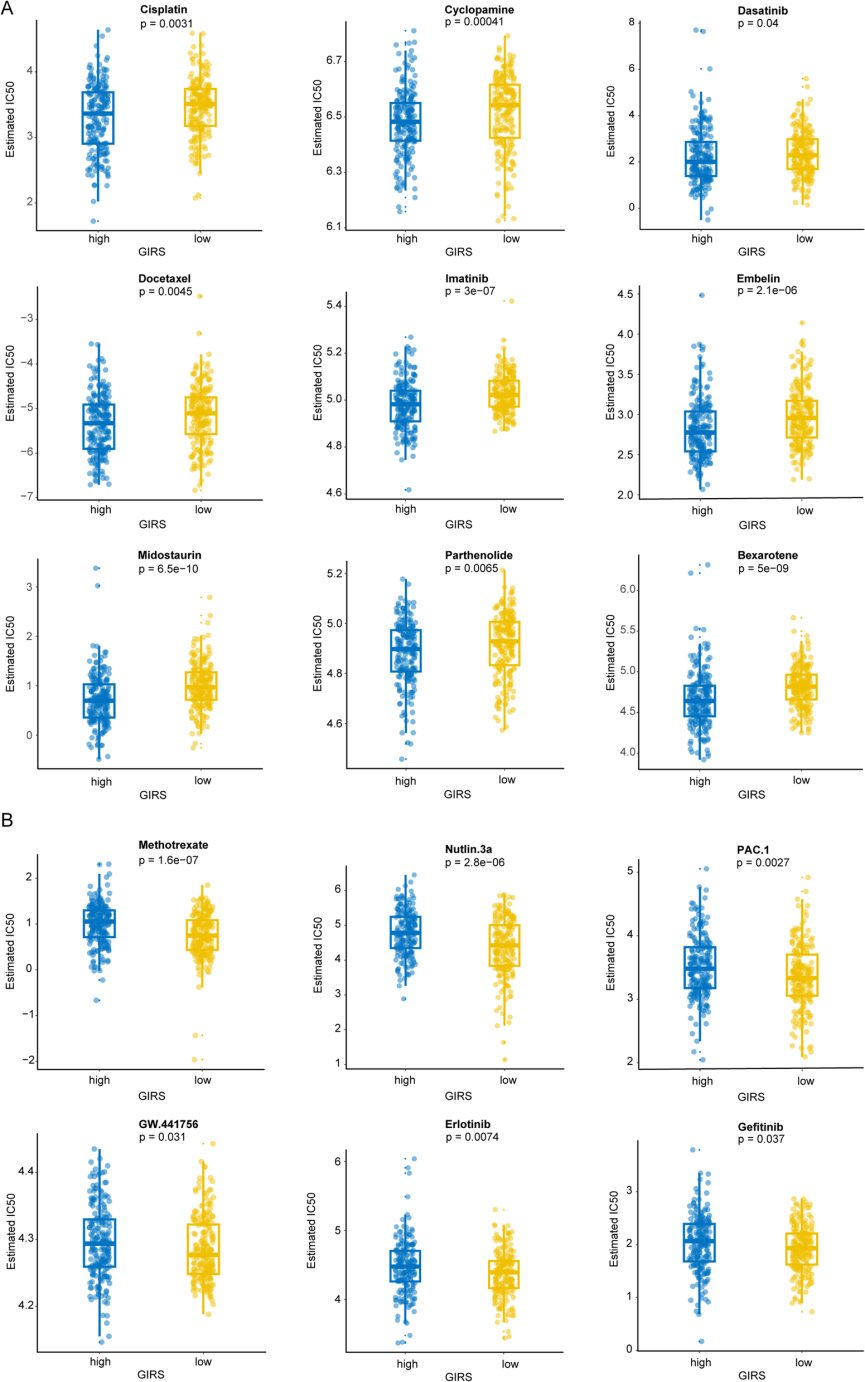
Drug sensitivity analysis in GIRS subgroups (**A**) Nine drugs that were more sensitive to high GIRS patients. **B** Six drugs that were more sensitive to low GIRS patients

The drug susceptibility analysis revealed that the high-risk group, as determined by GIRS, was more sensitive to Cisplatin, Cyclopamine, Dasatinib, Docetaxel, Imatinib, Embelin, Midostaurin, Parthenolide, and Bexarotene (Fig. 7A) while the low-risk group, was more sensitive to Nutlin, Methotrexate, PAC.1, GW.441756, Erlotinib, and Gefitinib (Fig. 7B). Remarkably, these findings aligned with a previous study to some extent [6], providing additional support for the significance and applicability of the gene signature in influencing medication decisions for BCa patients. Consequently, our gene signature held valuable potential for guiding the selection of chemotherapy and targeted agents for BCa patients.

To predict small-molecule targeted drugs specifically for the high-risk group, a differential expression analysis was performed between the high-risk and low-risk groups in the TCGA-BLCA cohort. Genes with a |log2FC|> 1 were considered differentially expressed using the R package “limma”. Subsequently, 150 upregulated DEGs and 150 downregulated DEGs were uploaded to the CMap database (https://portals.broadinstitute.org/cmap/). By analyzing the connectivity scores, which represent the similarity between the gene expression pattern induced by a drug and the gene expression profile of the high-risk group, the top ten small-molecule targeted drugs were identified. The specific list of these drugs can be found in Additional file 1: Table S3. In summary, the GIRS could provide valuable guidance for drug selection in BCa patients, enabling individualized precision drug therapy for different GIRS subgroups.

### Identification of prognostic hub gene and upstream transcription factors (TFs)

**Fig. 8:**
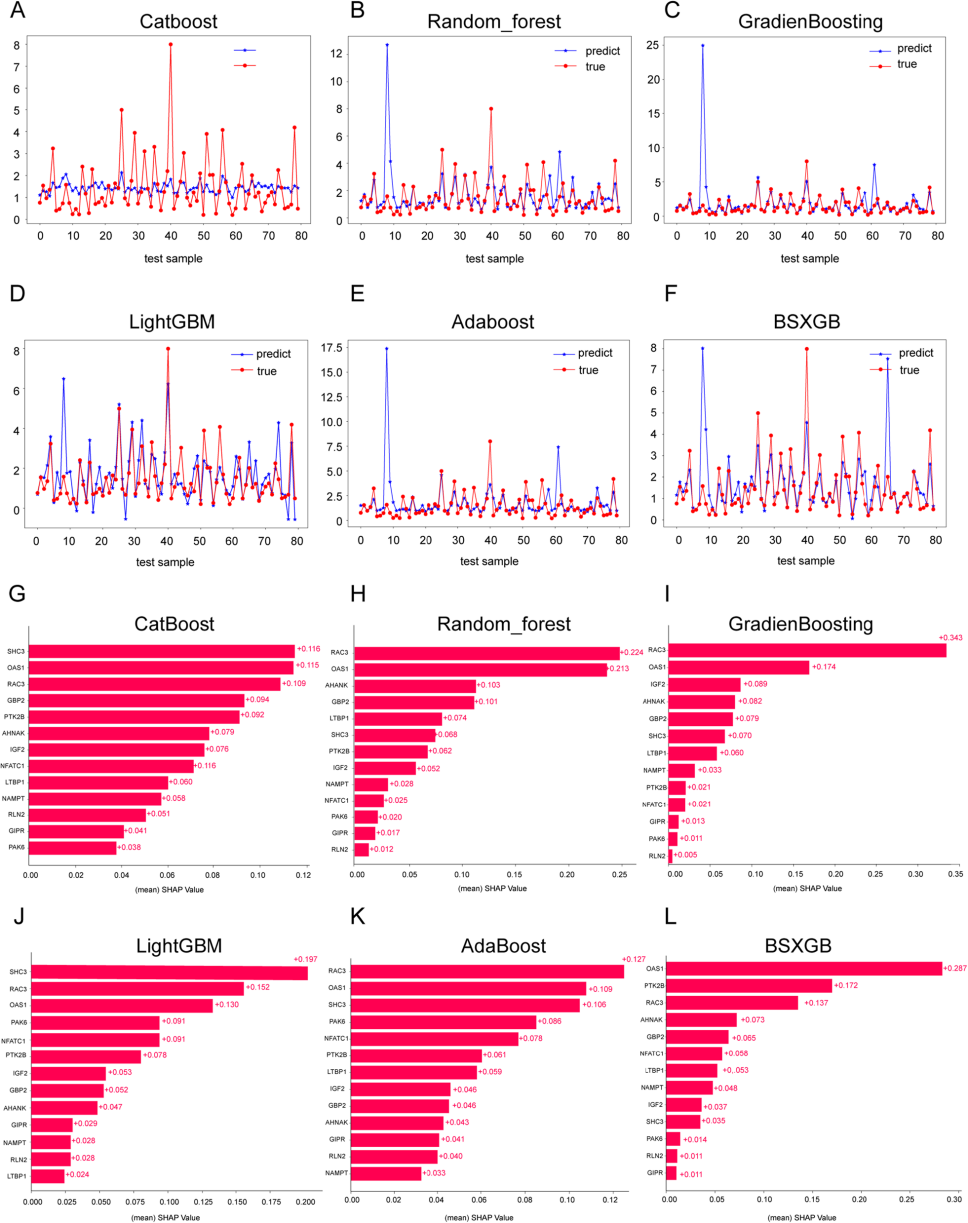
Hub gene identification by 6 machine learning algorithms. **A–F** 6 machine learning classifier accuracy (Catboost, Random Forest, GDBT, LGBM, Adaboost, and BSXGB). Red lines represent true data and blue lines represent predicted data. **G–L** Through six machine learning algorithms (Catboost, Random Forest, GDBT, LGBM, Adaboost, and BSXGB), the contribution value of each gene that makes up the signature to the model is calculated and ranked from largest to smallest. SHAP value represents the absolute average of the effect of each gene on the model

To identify the hub gene that significantly contributed to the predicted model, six machine-learning algorithms (XGboost, Catboost, Random Forest, AdaBoost, LightGBM, and GradienBoosting) were employed to analyze the feature importance of the 13 genes in the signature. As shown in Fig. 8A–F, the predictive accuracy of the six machine learning algorithms was notable. Among them, LightGBM algorithm demonstrated the highest accuracy in survival prediction, while CatBoost exhibited a relatively conservative prediction. Subsequently, we utilized SHAP values to interpret the contribution of each gene to the model. As illustrated in Fig. 8G–L, we observed that the SHAP values for RAC3 consistently ranked within the top three across the results of all six algorithms. 3 algorithms (Random Forest, GradienBoosting, AdaBoost) proved that RAC3 had the largest contribution to the model. In the other three algorithms (XGboost, Catboost, LightGBM), the contribution value of RAC3 to the model was ranked in second or third place. To sum up, the contribution of RAC3 was consistent and stable across different algorithms. Therefore, we designate RAC3 as the hub gene with the highest contribution to the model predictions.

Subsequently, we examined potential transcription factors responsible for RAC3 transcription by Cistrome DB Toolkit. As shown in Additional file 1: Fig. S6A and B, the top 10 transcription factors were ELF1, H2AZ1, NRF1, KDM2B, ZBTB7A, MYC, MYH11, SMC1A, POLR2A, and SAP30. Among these identified potential factors, the expression levels of H2AZ1 and NRF1 were positively associated with RAC3 (H2AZ1, R = 0.33, p = 1.6e−1, Additional file 1: Fig. S6C; NRF1, R = 0.32, p = 9.5e−11, Additional file 1: Fig. S6C). These results indicated that RAC3 was the hub gene in our predictive signature, and its transcription was most likely regulated by H2AZ1 and NRF1.

### Molecular docking analysis

**Fig. 9:**
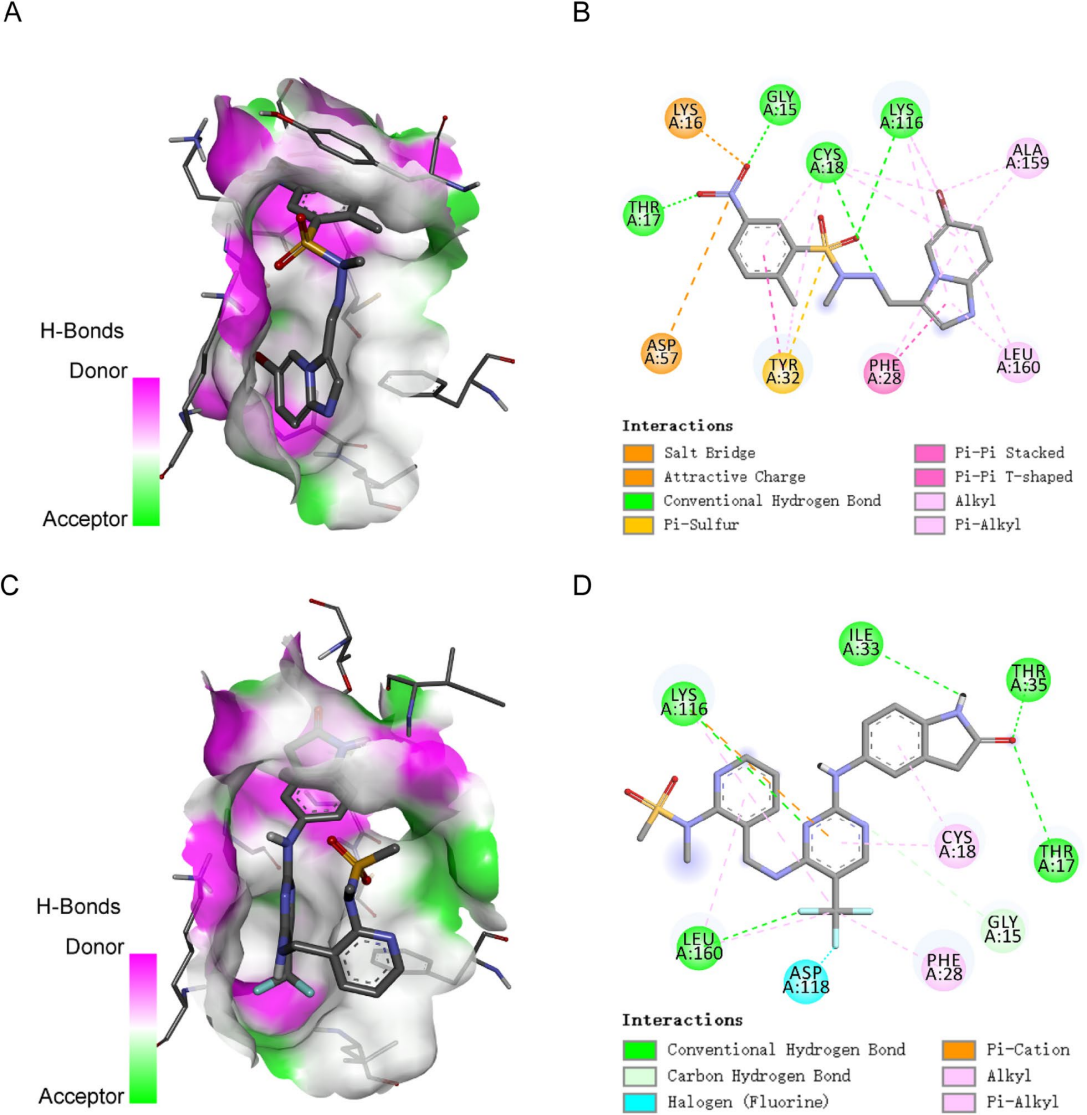
3D structure of RAC3- PIK-75 complex and RAC3- PF-562271 complex based on molecular docking. **A** The binding mode of the complex RAC3 with PIK-75. **B** The amino acid residues to which PIK-75 bonded in the protein pocket. **C** The binding mode of the complex RAC3 with PF-562271. **D** The amino acid residues to which PF-562271 bonded in the protein pocket

To further explore the most effective small-molecular drug targeting on RAC3 protein, we performed a molecular docking analysis of the RAC3 protein with the top ten screened small-molecular drugs (5-iodotubercidin, A-443644, AT-7519, Bisindolyl-aleimide-ix, CDK-inhibitor, Dactinomycin, JNK-9L, PF-562271, PIK75, Topoisomerase) sourced from CMap. The molecular docking results were presented in the data-heatmap file (Additional file 1: Table S4), and the visual representation is shown in Fig. 9. It was confirmable that binding energy serves as a reliable predictor of the binding activity between receptors and ligands. Actually, Lower binding energy values indicated tighter conformational binding. A binding energy below − 5 kcal/mol signified good binding ability, while a binding energy below − 7 kcal/mol suggested strong activity [7]. As depicted in Additional file 1: Table S4, all small-molecule drug compounds exhibited binding energies below − 6 kcal/mol, signifying a favorable match with the target protein, RAC3. Among them, two drugs, named PIK75 (binding energy = − 9.5 kcal/mol) and PF-562271 (binding energy = − 9.3 kcal/mol), demonstrated the lowest binding energies and were visualized using Pymol2.1 software (Fig. 9A and C). The amino acid residues to which the two drugs bonded in the protein pocket were clearly delineated in Fig. 9B and D. It was evident that PIK75 displayed the highest affinity for the active site of the RAC3 protein. The active amino acid residues of RAC3 that interacted to PIK75 included LYS-16, ASP-57, TYR-32, THR-17, GLY-15, CYS-18, LYS-116, PHE-28, LEU-160, and ALA-159 (Fig. 9B). PIK75 formed multiple hydrogen bonds with THR-17, GLY-15, CYS-18, and LYS-116, yielding a strong effect on binding stability. In addition, the benzene ring of PIK-75 formed robust π–π conjugated interactions with PHE-28, which contributed to a strong hydrophobic effect and facilitated the formation of the stable complex as well. Similarly, PF-562271 formed hydrogen bonds (LYS-116, ILE-33, THR-35, THR-17, and LEU-160), halogen bon (ASP-118) and hydrophobic (PH-28, CYS-18) interactions with RAC3 (Fig. 9D). These interactions effectively facilitated the formation of stable complexes between small molecules and the Rac3 protein. To sum up, Both PIK-75 and PF-562271 exhibited favorable interactions with RAC3 through various mechanisms and demonstrated strong associations with these protein targets.

### PIK-75 down-regulated the gene expression of RAC3 at mRNA and protein levels

**Fig. 10:**
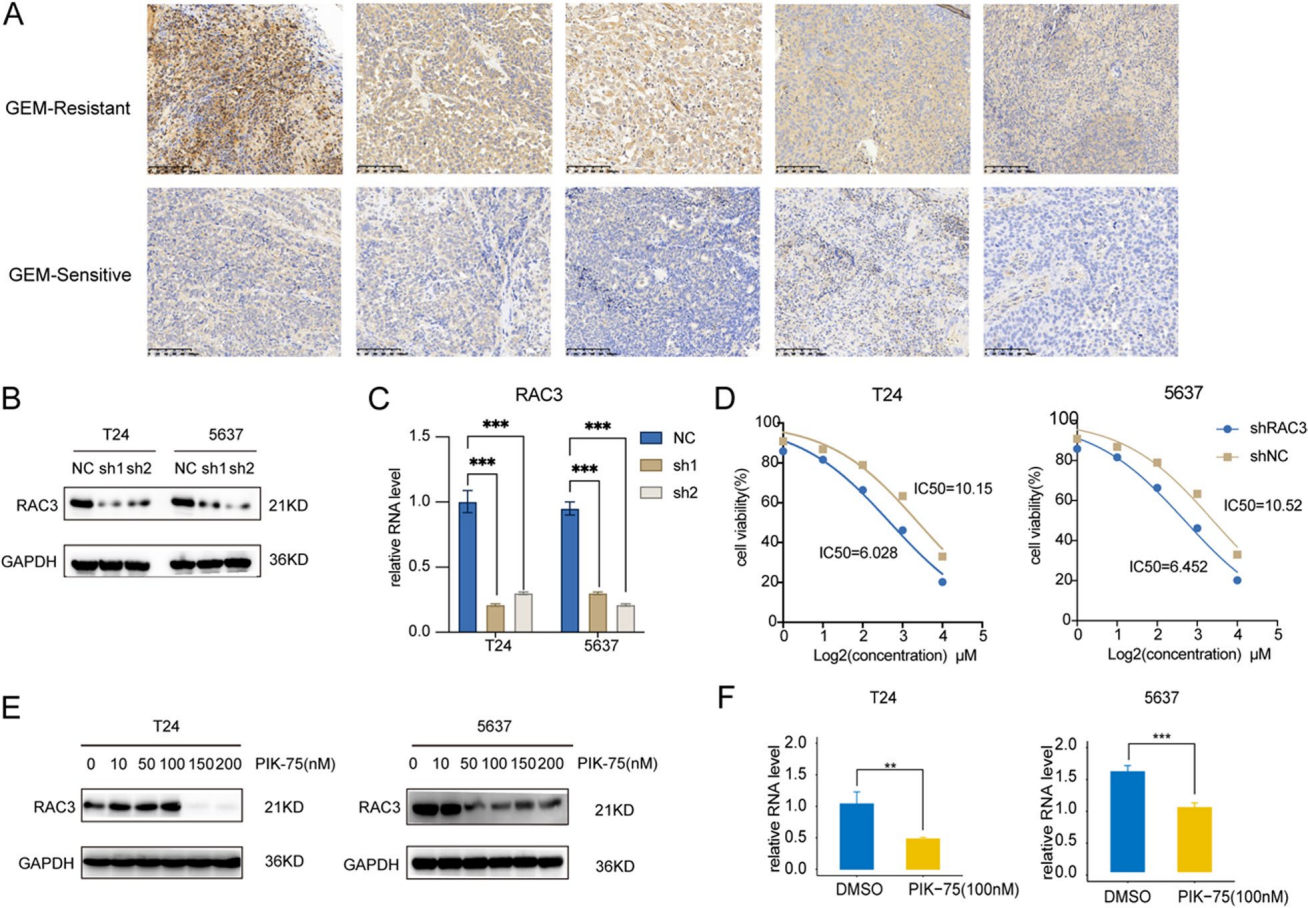
RAC3 was higher expressed in GEM-resistant patients. **A** Representative images of IHC staining of RAC3 of tumor tissue from gemcitabine-sensitive (GEM-sensitive) or gemcitabine-resistant (GEM-resistant) patients. **B** Western blot analysis of RAC3 in T24 and 5637 after transfected with shNC or shRAC3. **C** qPCR analysis of the mRNA levels of RAC3 after transfected with shNC or shRAC3. **D** IC50 analysis of T24 and 5637 treated by gemcitabine. **E** Western blot analysis of RAC3 after PIK-75- or DMSO-treated. **F** qPCR analysis of the mRNA levels of RAC3 after PIK-75- or DMSO-treated

Previous studies have shown that RAC3, a small GTP-binding protein belonging to the RAS superfamily, controls a variety of biological processes such as cell growth, cytoskeletal reorganization, and protein kinase activation [8]. Importantly, it served as an oncogene in a variety of tumors, including BCa, which was indicated closely associated with cancer invasion [9–11]. Cheng et al. [9] found that RAC3 expression was overexpressed in BCa tissues and cells, and patients with up-regulated RAC3 had worse survival outcomes. Wang et al. [12] found that RAC3 mediated autophagy through the PI3K/AKT/mTOR pathway and promoted the occurrence and metastasis of BCa. Our bioinformatics validation in public databases also showed that RAC3 was one of the upregulated oncogenes that negatively correlated with survival status in BCa, which was consistent with previous study [9, 12]. To further validate the relationship between RAC3 and gemcitabine sensitivity, we collected tumor tissue paraffin specimens from 20 patients with NMIBC who received intravesical gemcitabine instillation postoperatively. Among them, 10 patients exhibited gemcitabine resistance, experiencing tumor recurrence shortly, while the remaining 10 patients were gemcitabine-sensitive, with no recurrence within 2 years. IHC staining was conducted to assess the expression of RAC3. The results revealed a significant upregulation of RAC3 expression in gemcitabine-resistant patients compared to gemcitabine-sensitive patients (Fig. 10A, Additional file 1: Fig. S7A). This suggested that patients with elevated RAC3 expression may be more prone to developing gemcitabine resistance. Then, we knocked down the expression of RAC3 in T24 and 5637 cell lines. The efficiency was confirmed through Western blot (Fig. 10B) and quantitative PCR (qPCR) (Fig. 10C). Subsequently, we selected sh2, which exhibited a significant reduction in RAC3 expression, for further experiments. We found that the IC50 values for gemcitabine decreased from 10.15 μM to 6.028 μM in T24 and from 10.52 μM to 6.452 μM in 5637 cells after knocking down the expression of the RAC3 (Fig. 10D). We could indicate that inhibiting the expression of RAC3 may enhance the sensitivity of bladder cancer cells to gemcitabine.

### PIK-75 inhibited the proliferation, and migration and enhanced the apoptosis of bladder cancer cells and reduced the viability of bladder cancer organoids

**Fig.11:**
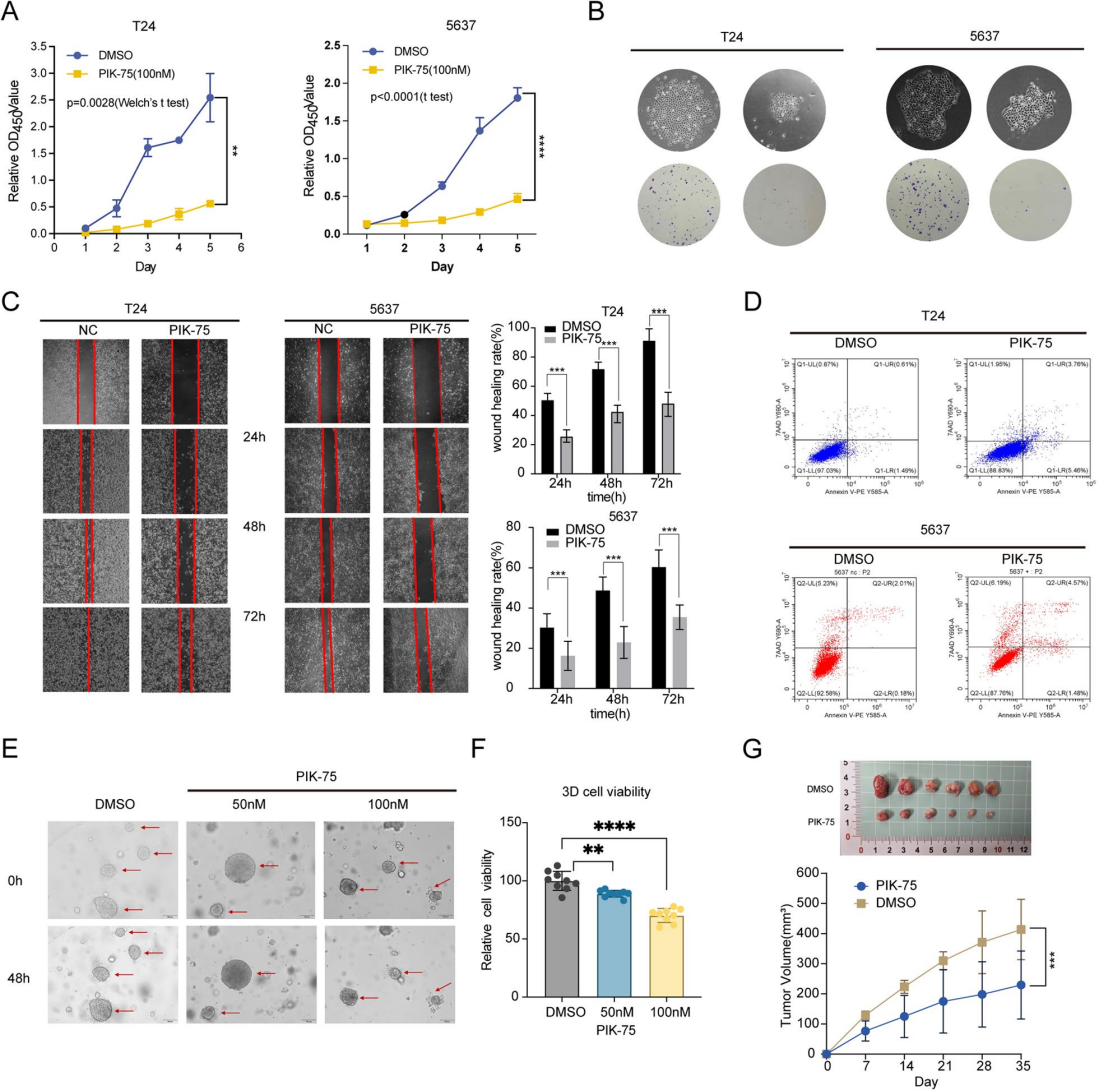
PIK-75 inhibit the growth of BCa in vitro and in vivo. **A, B** CCK8 assay (**A**) and colony formation assay (**B**) of BCa cells. **C** A wound-healing assay of BCa cells. **D** The FACS results of Annexin V-PE/7-AAD staining after PIK-75- or DMSO-treated. **E, F** Morphological changes (**E**) and 3D cell viability assay (**F**) of BCa organoids after PIK-75- or DMSO-treated. **G** Images of the xenograft tumors from MB49 cells subcutaneously injected c57BL/6 mice and tumor growth curves were plotted. The mice were subjected to intraperitoneal administration (i.p.) of PIK-75 or DMSO (as a control) following tumor establishment

Then, we delved into whether PIK-75 could influence the malignant biological behaviors exhibited by BCa cells. We initiated an assessment of our tumor cell lines' sensitivity to PIK-75. Notably, the Bladder Cancer cell lines T24 and 5637 displayed nearly equivalent drug sensitivity and tolerance to PIK-75, with IC50 values of 122.8 nM and 131.6 nM, respectively (Additional file 1: Fig. S7B). Notably, upon treatment with PIK-75 (100 nM) for 24 h, the survival rate of both cell lines exceeded 70–80%. Additionally, there was a significant reduction in RAC3 expression observed at both mRNA and protein levels. Particularly noteworthy was the T24 cell line, where the RAC3 protein became nearly undetectable after treatment (Fig. 10E, F). CCK-8 assay further verified the proliferation inhibition of BCa cells by PIK-75, which revealed a statistically significant lower OD450 value at all time points (Fig. 11A). Furthermore, the results of the cloning formation experiment showed that regardless of the colony formation numbers or size, the PIK-75-treated group was significantly less than the control group. The colony formation efficiency was dramatically decreased in vitro (Fig. 11B). The results of the wound healing assay were shown in Fig. 11C. The cells in the control group migrated and nearly covered the scratch wound rapidly in 72 h, while the migration ability of tumor cells was inhibited after incubation with PIK-75(100 nM). The FACS analysis of Annexin V-PE/7-AAD staining demonstrated an increase in the early apoptosis rate of the T24 cell line to approximately 5–6%, with a concurrent rise in the late apoptosis/necrosis rate to about 3–4%. Additionally, the early apoptosis rate of 5637 cells increased to around 2%, accompanied by a rise in the late apoptosis/necrosis rate to approximately 5–6% (Fig. 11D). Furthermore, treatment of BCa organoids with PIK-75 or DMSO for 48 h resulted in a significant 30% reduction in cell viability, as illustrated in Fig. 11E and F). Following this, we further validated the efficacy of PIK-75 in a subcutaneous tumor model in mice. The results demonstrated that mice treated with PIK-75 exhibited slower tumor growth and smaller tumor volumes compared to the control group (Fig. 11G). These findings highlight the potential therapeutic efficacy of PIK-75 for BCa.

## Discussion

BCa poses a significant social health challenge due to its sustained high morbidity and mortality rates, and unfortunately, its burden has remained largely unchanged over the course of several decades [13]. Gemcitabine (GEM) has become the cornerstone of chemotherapy for BCa, while the persistent hurdles of chemoresistance and tumor relapse continue to pose major clinical challenges. Presently, clinicians rely on the TNM staging system as the basis for treatment decisions and surveillance strategies [14]. However, this system primarily employs a macro staging approach based on anatomical considerations and fails to account for genomic alterations. Consequently, there exist substantial limitations in the selection of appropriate treatment strategies for patients with chemotherapeutic-resistant BCa, resulting in unfavorable survival outcomes and prognoses. Therefore, there is an urgent imperative to establish a more robust personalized assessment protocol capable of identifying patients who may exhibit refractory BCa and predicting their likelihood of poor survival prognosis. To date, numerous independent studies have focused extensively on gene signatures or novel biomarkers to enhance the accuracy of predicting GEM chemosensitivity and patient prognosis [15, 16].

Nonetheless, existing gene signatures or biomarkers correlated with chemoresistance at the transcription level are promising but have not yet been suitable for routine clinical applications. Moreover, descriptions of changes occurring in the immune TME before and after chemotherapy are lacking [1]. To address the demands of the clinical decision support system, here we aimed to investigate the regularity of genomic and transcriptome alterations of GEM-resistant BCa cell lines from the GSE190636 dataset. Additionally, we explored the combination pattern of chemo-immune features (gene signature), in an attempt to identify the driving factors behind tumor treatment resistance. Our objective was to stratify patients into different risk groups, thereby providing a novel perspective for personalized therapy and prognosis prediction. To rationalize the changes in the immune TME induced by chemotherapy, GSE190636, a transcriptomic data of GEM-resistant or sensitive BCa cell lines, was used to obtain DEGs, and further intersected with a prior defined immune-related gene set, IRGs, to define GIRGs. Next, we adopted the a stepwise survival analysis framework, incorporating the uni-Cox, LASSO and multi-Cox survival analysis to identify GIRGs. Subsequently, a 13-gene signature, GIRS, was constructed using multiple well-established public BCa patient cohorts, along with a nomogram incorporating GIRS and other clinical factors. Notably, in previous studies, when screening for prognosis, univariate Cox analysis was usually conducted to screen out relevant variables, followed by multivariate Cox analysis to further confirm the independent association of these variables with survival. However, this approach does not consider the influence of multicollinearity among variables. Most signatures perform well on their training queues and a few external cohorts but exhibit weak performance in new cohorts [17], probably due to poor generalization caused by overfitting. The LASSO algorithm addresses this issue by simultaneously achieving variable selection and model parameter estimation, effectively solving the problem of multicollinearity in regression analysis and mitigating overfitting concerns to a certain extent [18]. The stepwise modeling approach of uni- Cox + LASSO + multi-Cox enables a more precise estimation of each gene's impact on survival time. This enhances the accuracy and interpretability of our prognostic signature, while preventing overfitting and improving model generalizability. Indeed, Our GIRS signature and nomogram demonstrated moderate to high accuracy and stable performance in multiple independent cohorts, as evidenced by Kaplan–Meier analysis, ROC curves, C-index, calibration curves, decision curves, and subgroup analysis. These results highlight the potential of our model to significantly enhance medical practice. Notably, in the validation cohort, GSE32894, the areas under the ROC curves of our novel nomogram exceeded 0.9, and the C-index reached 0.9265, which indicates high accuracy and strong extrapolation of our model. In the subgroup analysis of GSE32894 (Fig. 6E), we also found that the risk stratification according to GIRS was in alignment with the result of a previous research [19], where higher GIRS corresponded to subgroups with worse prognosis (MS2b2.1, MS2b2.2) or higher T stage, confirming the sufficient reliability of our signature.

Cancer immunotherapy represents a revolutionary change in the therapeutic landscape for solid tumors, particularly bringing great therapeutic benefits for advanced disease [20, 21]. However, only a small percentage of BCa patients are responsive to ICIs, delaying disease progression and improving survival. In addition, certain emerging chemo-immune synergistic combination regimens, such as pembrolizumab + GEM/CDDP (KEYNOTE-361), have failed to demonstrate significant advantages over chemotherapy alone in BCa [22, 23]. In this study, by analyzing multiple cohorts with drug treatment information (GSE91061; IMvigor410; GSE52219), we also identified the clinical values of GIRS in the prediction of drug sensitivity and efficacy. Furthermore, we extensively explored the immune landscape at the bioinformatics level through immune infiltration analysis, GSVA enrichment analysis, and correlation analysis. Encouragingly, the GIRS classification of patients aligned with the actual regression of patients following immune checkpoint inhibitors (ICIs) administration. Patients who achieved complete or partial remission (CR/PR) generally exhibited lower GIRS values. Among those classified as low risk by GIRS, a higher proportion of individuals responded to MVAC neoadjuvant chemotherapy regimens.

Moreover, based on the preliminary immune infiltration analysis by the CIBERSORT algorithm and correlation analysis, GIRS showed a positive correlation with activated CD4 memory T cells and macrophage M1. Consequently, we conducted further analysis on the differential infiltration of 30 types of T cells and macrophages in the high and low GIRS groups. The results indicated that patients in the low GIRS group demonstrated overall higher levels of T cell and macrophage infiltration. Increased infiltration of immune cells may enhance anti-tumor immunity and lead to improved immunotherapeutic outcomes. However, it is important to note that due to limitations in the available data, some of the comparisons between the two groups only exhibited a trend without statistical significance. Therefore, further validation is required to support these findings.

Precision medicine has the objective to individualize medical treatment for each patient as early as possible. Therefore, we utilized the cMAP database to select chemotherapeutic or targeted agents that showed differential activity in different GIRS subgroups. This information could serve as a valuable reference for clinicians to formulate individualized therapeutic strategies in the future. Collectively, these findings suggest that our GIRS signature and nomogram have the potential to provide reasonable guidance for identifying treatment-sensitive BCa patients receiving first-line immunotherapy, chemotherapy, or targeted therapy. In conclusion, our study supports the notion that the application of our GIRS signature and nomogram can aid in the individualization of treatment approaches for BCa patients. By providing reasonable guidance for the selection of appropriate therapeutic interventions, including immunotherapy, chemotherapy, or targeted therapy, our findings facilitate the advancement of precision medicine.

As displayed in the forest plot in Fig. 2D, the genes that constituted our signature are as follows: OAS1, AHNAK, LTBP1, RAC3, GBP2, SHC3, NFATC1, GIPR, PTK2B, PAK6, RLN2, NAMPT, and IGF2. Numerous previous studies have described the possible functions of some of these genes. Gu et al. indicated that AHNAK acted as a tumor suppressor to assist p53 in inhibiting the transcription of stemness-related genes [24]. Lee et al. identified AHNAK as a novel candidate biomarker for the liquid-based cytological diagnosis of bladder uroepithelial carcinoma through quantitative proteomics studies [25]. Kawahara et al. study found that NFATc1 was instrumental in BCa growth and that NFATc1 inactivation, especially with CsA and FK506, poses a potential treatment for BCa [26]. Additionally, Cheng et al. demonstrated that RAC3 played a carcinogenic role in BCa cells, and overexpression of RAC3 activated JAK/STAT oncogenic signaling pathway [9, 27]. Wang et al. found that knocking down RAC3 enhanced autophagy mediated by PI3K/AKT/mTOR pathway. Targeting RAC3 was a promising therapeutic method to inhibit BCa progression and prolong survival time [12]. Next, we employed machine-learning approaches to filtering out the hub gene that made the most significant contribution to the GIRS signature. In the field of machine learning, a fundamental theorem asserts that there is no algorithm capable of perfectly solving all problems, especially in the context of supervised learning, such as predictive modeling. These algorithms are influenced by various factors. So, it's recommended to compare and validate results when using multiple algorithms to ensure consistent and reliable outcomes. In this study, we employed a total of six different machine learning algorithms to analyze the feature importance of the 13 genes in the signature to augment the confidence and stability of our results. Ultimately, the contribution of RAC3 was consistent and stable across different algorithms. Therefore, we designated RAC3 as the hub gene with the highest contribution to the model predictions. We validated the expression of RAC3 in tissue specimens from 20 NMIBC patients who received intravesical gemcitabine instillation. We observed higher levels of RAC3 expression in gemcitabine-resistant patients compared to gemcitabine-sensitive patients. In line with this, our in vitro findings demonstrated that knocking down RAC3 in T24 and 5637 cells led to an enhancement in their sensitivity to gemcitabine. This suggested that gemcitabine may not be recommended to be preferred as a postoperative perfusion drug in NMIBC patients with high RAC3 expression in clinical applications. However, the role of RAC3 in gemcitabine resistance required further validation through additional in vivo experiments in future studies. Additionally, we utilized molecular docking to identify the small molecule drug PIK-75 with the highest affinity for the hub gene RAC3. Notably, PIK-75 exhibited a significant influence on various malignant biological traits of BCa in vitro and in vivo. These findings highlight the potential therapeutic efficacy of PIK-75 for BCa.

By amalgamating machine learning, molecular docking, TF analysis, expression validation, functional assays, and IHC validation, our study has elucidated the potential role of RAC3 in gemcitabine resistance and BCa progression. In forthcoming research, we are committed to meticulously establishing gemcitabine-resistant cell lines to augment the comprehensiveness and persuasiveness of our findings. Crucially, our investigation identified PIK-75, a small molecule drug with a pronounced affinity for the hub gene, presenting it as a potential therapeutic option for BCa patients exhibiting insensitivity to gemcitabine. These findings create new avenues for further investigation and offer potential directions for the advancement of targeted therapies in BCa.

In conclusion, our study provides valuable insights and forms a foundation for future investigations in the dynamic landscape of BCa management. Through the implementation of GIRS to categorize patients into high and low-risk groups, our model serves as a crucial tool for clinicians, facilitating the formulation of personalized and effective treatment strategies.

## Conclusions

In conclusion, the GIRS prognostic signature, integrating immunological features and chemotherapy response, introduces an innovative approach to risk stratification in BCa patients. This signature can potentially improve prognostic prediction accuracy and guide the selection of individualized treatment regimens. By integrating immunological features and chemotherapy response, the GIRS signature addresses an important gap in risk assessment for BCa patients. It holds promise for enhancing clinical decision-making and optimizing treatment strategies. The hub gene, RAC3, plays a crucial role in BCa and is significantly associated with resistance to gemcitabine. This finding provides new clues for a deeper understanding of the mechanisms underlying BCa drug resistance and may serve as a key target in the search for treatment strategies. Concurrently, we identified that the small-molecule drug PIK-75 exhibits an affinity for RAC3, effectively targeting this pivotal gene. This lends robust support to the development of innovative treatment approaches, with PIK-75 demonstrating potential efficacy and clinical application prospects in BCa.

### Supplementary Information

**Additional file 1:** Supplementary Figures S1–S7 and Supplementary Tables S1–S5.

## Data Availability

This study analyzed publicly available data sets as described in the Method section. This data can be retrieved from here: UCSC Xena and GEO database, and these web links or unique identifiers for public cohorts are described in the paper. Source codes and supplementary data adopted to generate the results were deposited on supplementary materials.

## Abbreviations

BCa: Bladder cancer
GEM: Gemcitabine
NMIBC: Non-muscle invasive bladder cancer
TME: Tumor microenvironments
TNM: Tumor node metastasis
GIRS: Gemcitabine-based immune-related risk score
IRGs: Immune-related genes
DEGs: Differentially-expressed genes
GIRGs: Gemcitabine-based immune-related genes
TCGA: The cancer genome atlas
GEO: Gene expression omnibus
FUSCC: Fudan university shanghai cancer center
TPM: Transcript per kilobase million
CNA: Copy number variations
SNV: Single nucleotide variant
LASSO: Least absolute shrinkage and selection operator
K–M: Kaplan–Meier
CC: Consensus clustering
NMF: Nonnegative-matrix factorization
SNFCC+: Similarity network fusion plus consensus clustering
CDF: Cumulative distribution function
ssGSEA: Single sample gene set enrichment analysis
NES: Normalized enrichment score
GSVA: Gene set variation analysis
GSEA: Gene set enrichment analysis
TME: Tumor microenvironment
IC50: Semi-inhibitory concentration
LASSO: Least absolute shrinkage and selection operator
C-index: Concordance index
PD: Progressive disease
SD: Stable disease
CR: Complete response
PR: Partial response
Time-ROC: Time-dependent receiver operating characteristic
DCA: Decision curve analysis
CMap: The connectivity map
ICIs: Immune checkpoint inhibitors
TFs: Transcription factors
MVAC: Methotrexate + vinblastine sulfate + adriamycin + cisplatin
