# Supplementary figures and images for: Deciphering the prognostic features of bladder cancer through gemcitabine resistance and immune-related gene analysis and identifying potential small molecular drug PIK-75
Source: Cancer Cell Int. 2024 Apr 3;24:125. doi: 10.1186/s12935-024-03258-9 (PMC10993528; doi:10.1186/s12935-024-03258-9)

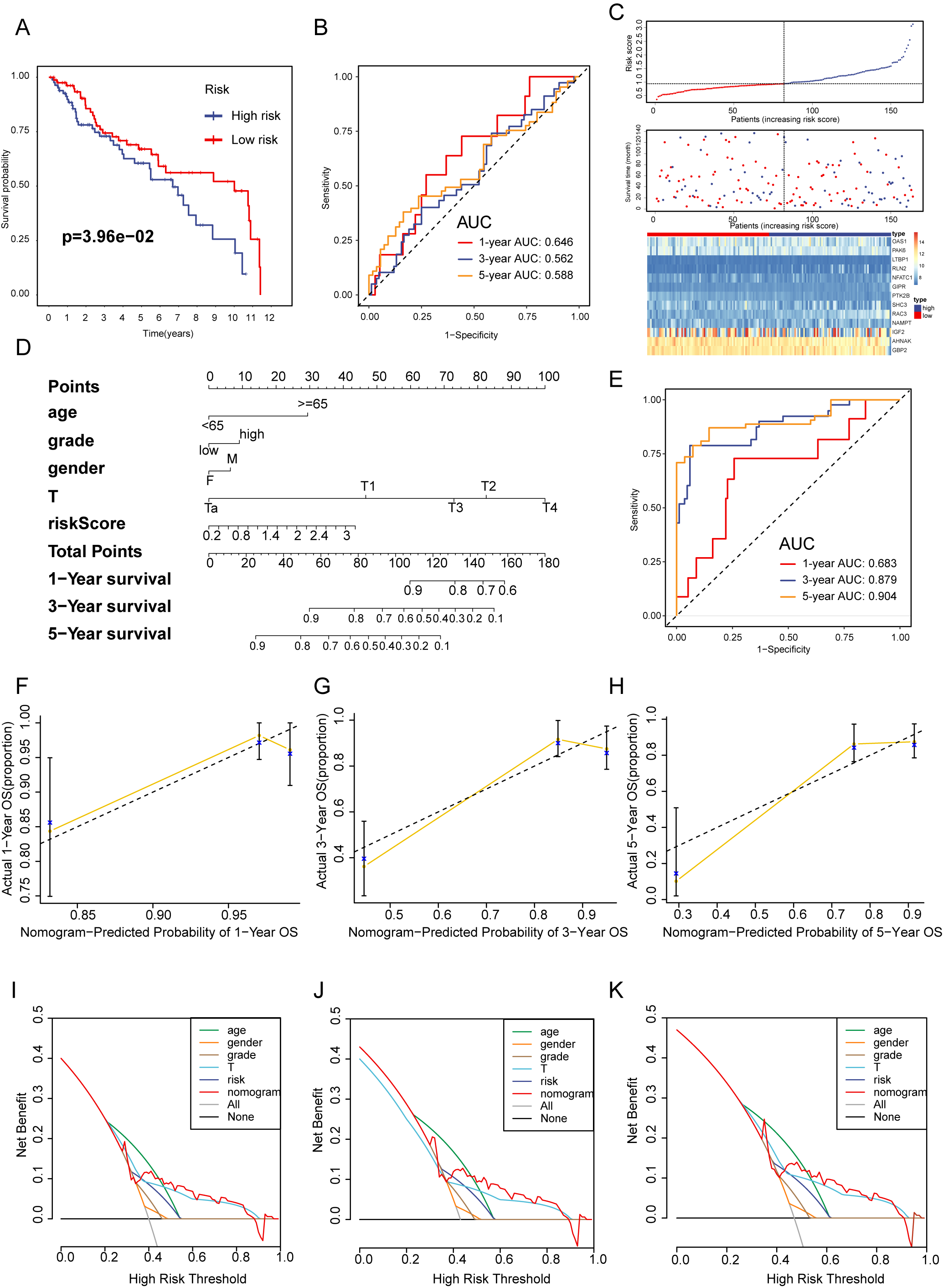

Supplement: Supplementary file 1 — Additional file 1: Supplementary Figures S1–S7 and Supplementary Tables S1–S5. [file 12935_2024_3258_MOESM1_ESM.zip › New folder/S1.tif]

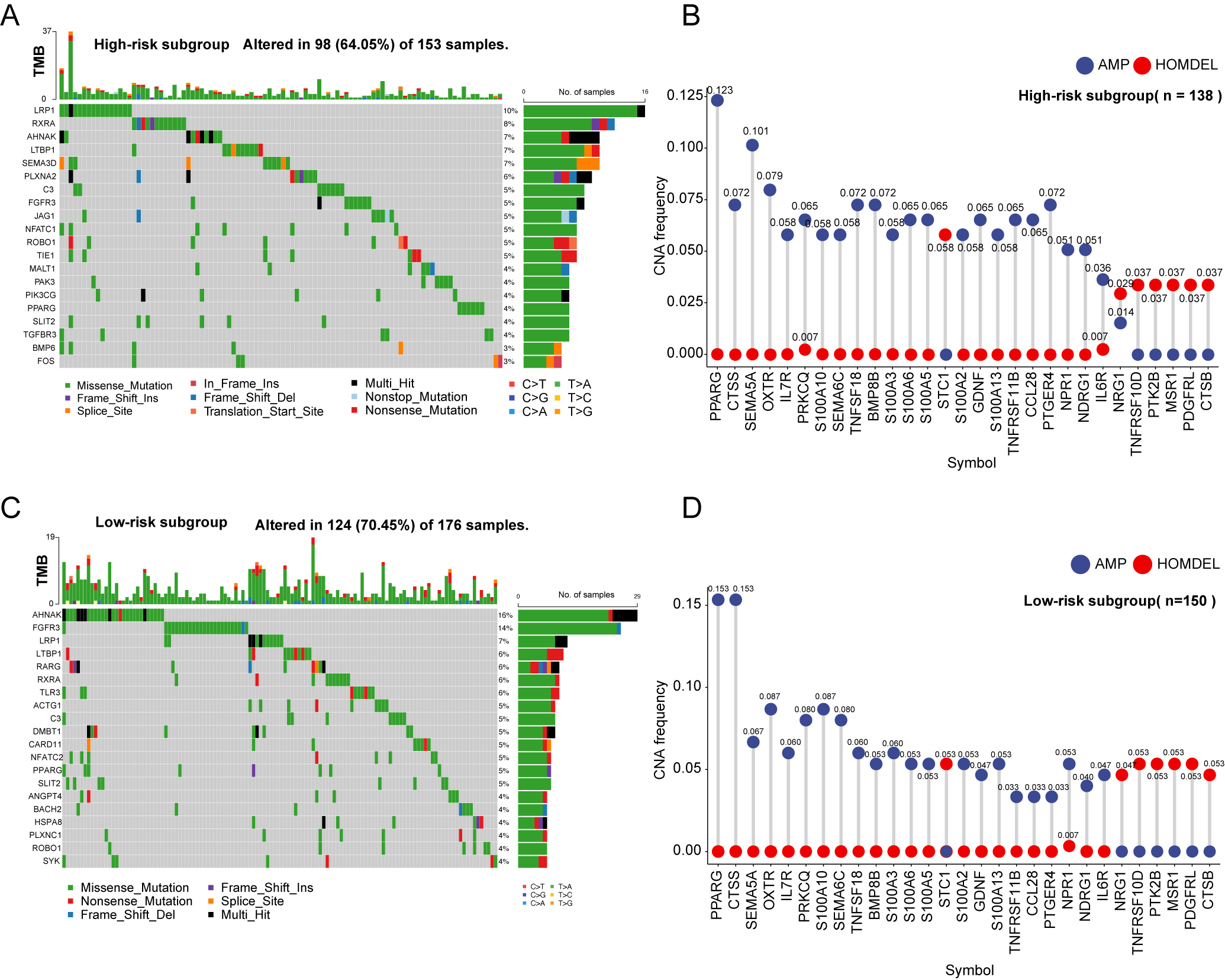

Supplement: Supplementary file 1 — Additional file 1: Supplementary Figures S1–S7 and Supplementary Tables S1–S5. [file 12935_2024_3258_MOESM1_ESM.zip › New folder/S2.tif]

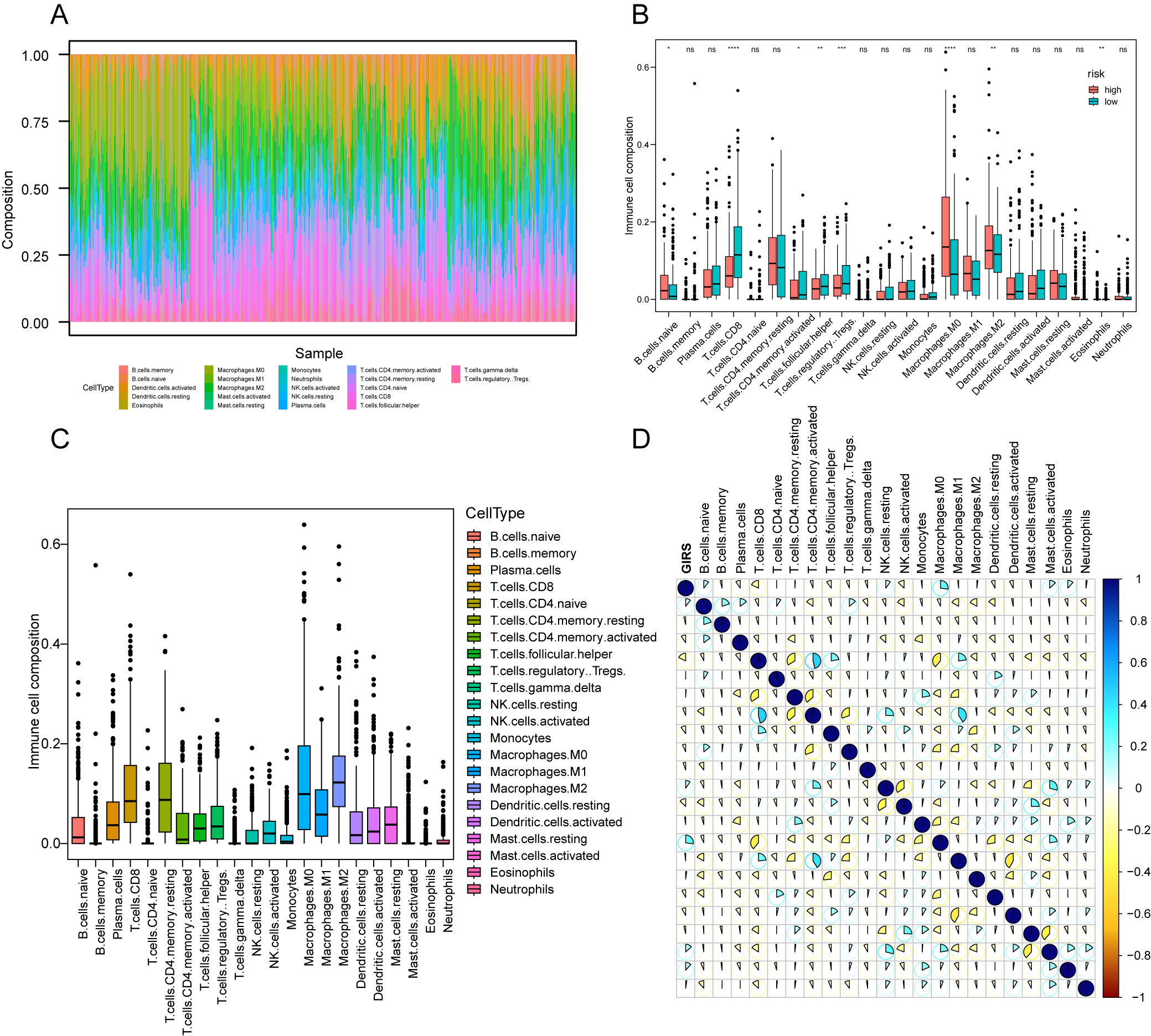

Supplement: Supplementary file 1 — Additional file 1: Supplementary Figures S1–S7 and Supplementary Tables S1–S5. [file 12935_2024_3258_MOESM1_ESM.zip › New folder/S3.tif]

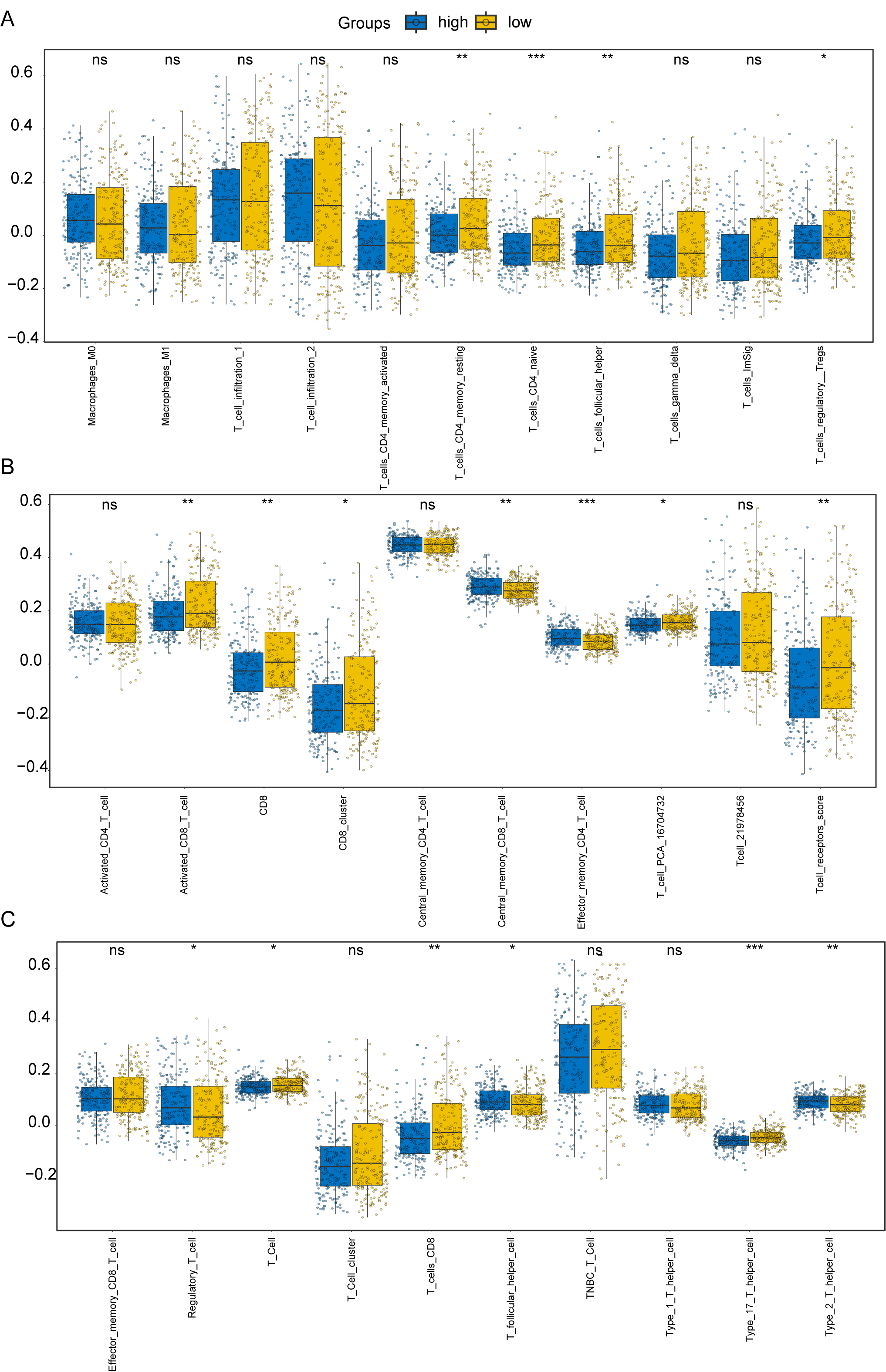

Supplement: Supplementary file 1 — Additional file 1: Supplementary Figures S1–S7 and Supplementary Tables S1–S5. [file 12935_2024_3258_MOESM1_ESM.zip › New folder/S4.tif]

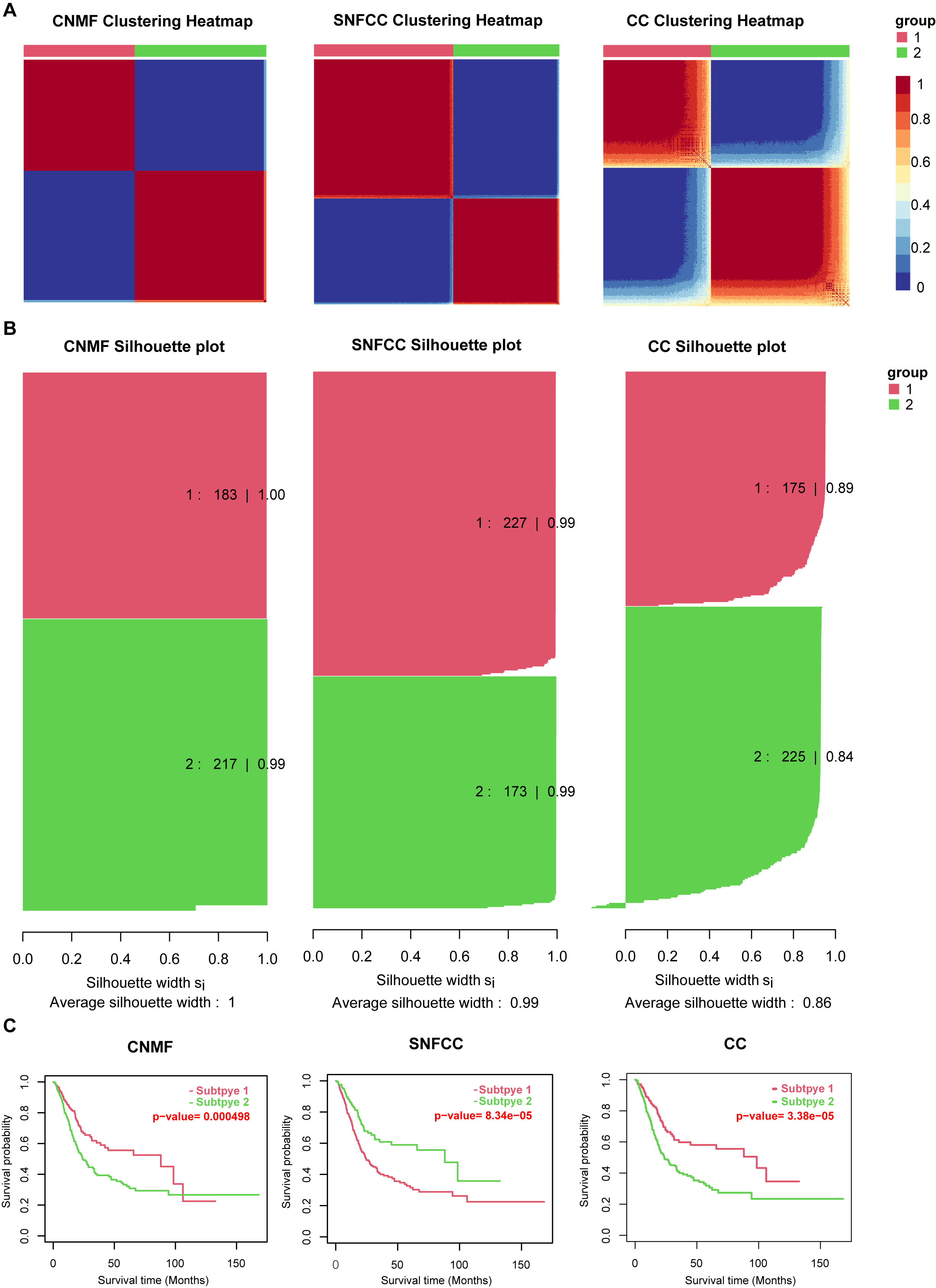

Supplement: Supplementary file 1 — Additional file 1: Supplementary Figures S1–S7 and Supplementary Tables S1–S5. [file 12935_2024_3258_MOESM1_ESM.zip › New folder/S5.tif]

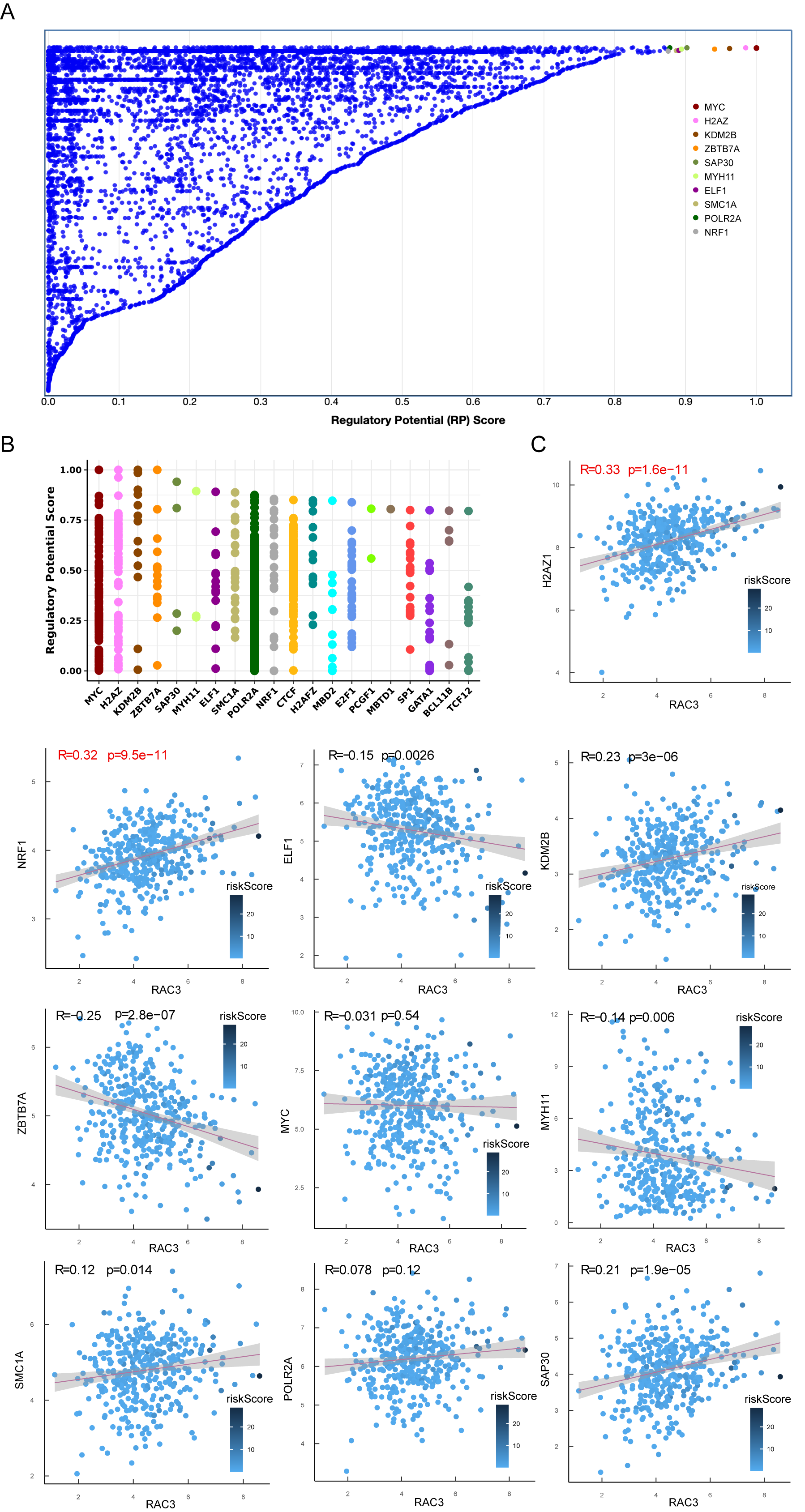

Supplement: Supplementary file 1 — Additional file 1: Supplementary Figures S1–S7 and Supplementary Tables S1–S5. [file 12935_2024_3258_MOESM1_ESM.zip › New folder/S6.tif]

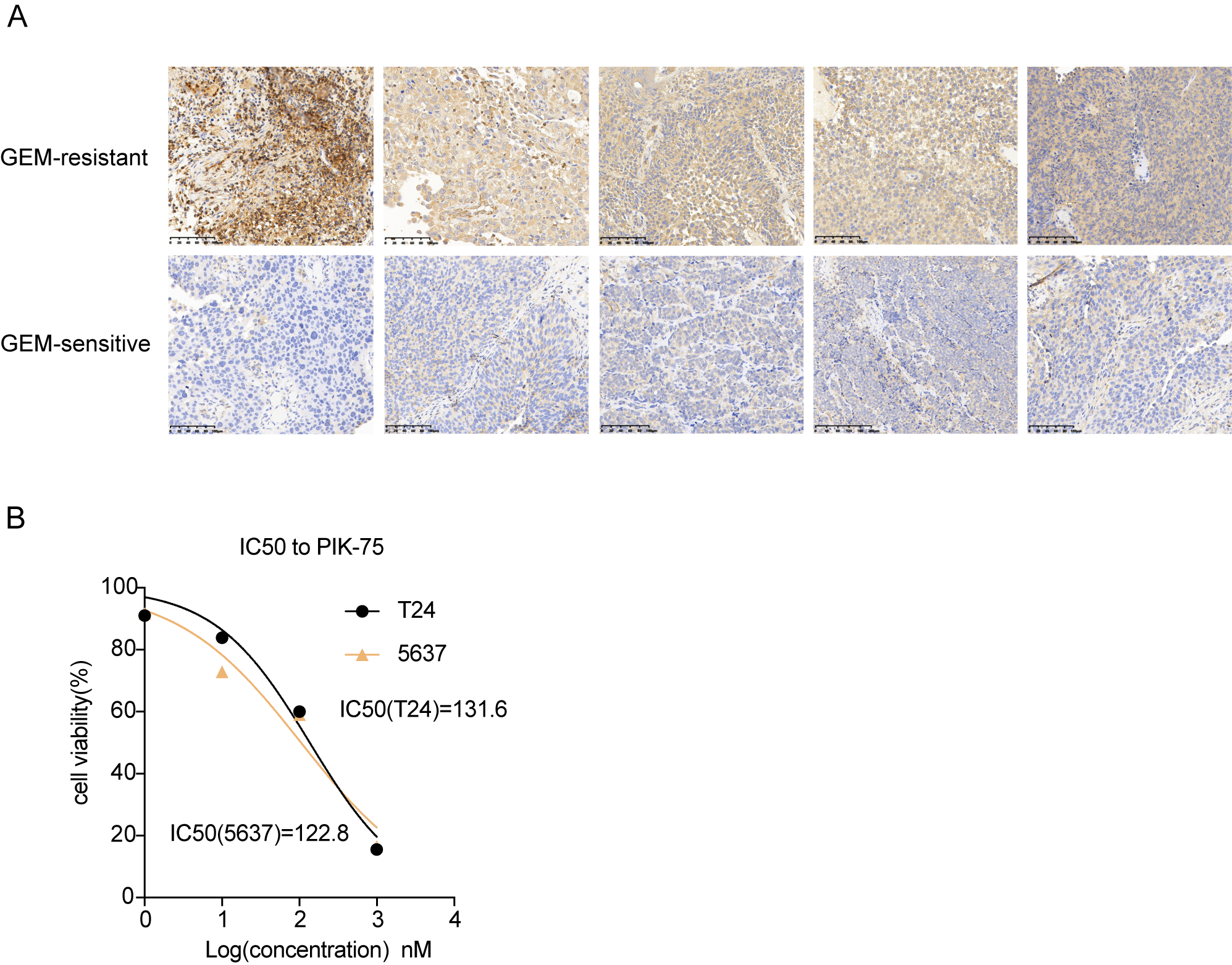

Supplement: Supplementary file 1 — Additional file 1: Supplementary Figures S1–S7 and Supplementary Tables S1–S5. [file 12935_2024_3258_MOESM1_ESM.zip › New folder/S7.tif]
